# Bacteriophages Control Epiphytic *Pseudomonas syringae* Populations in Highbush Blueberry Leaves

**DOI:** 10.1111/1751-7915.70438

**Published:** 2026-09-08

**Authors:** Cassidy Ball, Philip Lauman, Tongzhou Xu, Thomas Guy, Kasia Dadej, Robin Richter, Mark Lubberts, Keiran Cross, Meilin Ren, Someshwar Ravnit Latchman, Rishi Burlakoti, Xiangyu Deng, Karen Fong, Siyun Wang

**Affiliations:** ^1^ Faculty of Land and Food Systems The University of British Columbia Vancouver British Columbia Canada; ^2^ College of Agricultural and Environmental Sciences University of Georgia Griffin Georgia USA; ^3^ Summerland Research and Development Centre Agriculture & Agri‐Food Canada Summerland British Columbia Canada; ^4^ Ottawa Research and Development Centre Agriculture & Agri‐Food Canada Ottawa Ontario Canada; ^5^ Agassiz Research and Development Centre Agriculture & Agri‐Food Canada Agassiz British Columbia Canada

**Keywords:** agricultural biotechnology, jumbo phage, phage biocontrol, phage cocktail, phytopathogen, *Pseudomonas syringae* complex, *Vaccinium corymbosum*

## Abstract

The *Pseudomonas syringae* complex (Psc) is a group of globally distributed phytopathogens responsible for substantial agricultural losses. Although bacteriophage‐based biocontrol has shown promise against Psc, no studies have examined phages targeting blueberry‐tropic Psc lineages. Here, we isolated phages infecting Psc strains from diseased highbush blueberry ( *Vaccinium corymbosum* ), and evaluated their suitability for biocontrol using a multi‐stage screening pipeline incorporating host‐range analysis, comparative genomics, environmental stability testing, in vitro antibacterial efficacy assays and ex planta validation. Twelve of the isolated phages exhibited favourable host‐range characteristics. Genomic analyses revealed substantial phylogenetic diversity among these candidates but simultaneously identified multiple clonal groups, reducing the collection to eight non‐redundant phages spanning five distinct genera. Candidate phages generally retained infectivity under environmentally relevant conditions and exhibited heterogeneous but largely favourable stability profiles. Planktonic killing assays uncovered considerable variation in antibacterial efficacy, but phage performance appeared to be driven by infection compatibility and host‐specific factors rather than properties intrinsic to individual phages. Notably, the jumbo phage φCB10 emerged as a particularly promising candidate due to its strong antibacterial activity (median GRC = 0.943), favourable environmental stability and unique genomic features. Cocktails containing the most effective candidates produced substantial and longitudinally sustained reductions in epiphytic colonization of detached blueberry leaves by Psc, exceeding five orders of magnitude at peak efficacy and demonstrating robust activity in a biologically relevant ex planta system. Importantly, in vitro antibacterial efficacy was predictive of performance in our ex planta model (*r* = 0.67; *p* = 0.0003), supporting the utility of tiered screening approaches for candidate selection. Taken together, these findings establish a framework for the systematic identification and evaluation of phages targeting Psc, and support the development of phage‐based interventions for managing plant diseases.

## Graphical abstract

The *Pseudomonas syringae* complex (Psc) is a group of globally distributed phytopathogens responsible for substantial agricultural losses. Here, we established a framework for the systematic identification and evaluation of bacteriophages targeting Psc strains isolated from highbush blueberries and identified candidate phages capable of suppressing epiphytic Psc colonization of blueberry leaves, thereby supporting the development of phage‐based interventions for managing plant diseases.

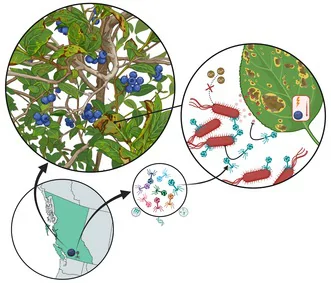

## 1 Introduction

The *Pseudomonas syringae* complex (Psc), a phylogenetic group comprising at least 15 bacterial species and more than 60 distinct pathovars, is among the most problematic bacterial phytopathogens worldwide. Collectively, these host‐specialized pathogens are known to cause disease in over 180 plant species, including virtually all economically relevant crops (Hwang et al. 2005; Gomila et al. 2017; Xin et al. 2018; Rabiey et al. 2020). The resulting reductions in yield, occasionally exacerbated by regional epidemics, can have substantial economic consequences and disrupt international supply chains—thereby posing a direct threat to global food security. Despite growing interest in the development of biocontrol strategies targeting Psc infections in a variety of crops (D'aes et al. 2010; Xin et al. 2018; Córdova et al. 2023; Holtappels et al. 2024; Giovanardi et al. 2025), no studies have yet examined their use in blueberries.

Blueberries are a major agricultural commodity, with global production reaching roughly 2.14 × 10^9^ kg in 2024 (International Blueberry Organization 2025). Canada is the fourth‐largest producer worldwide, accounting for nearly one‐sixth of global output, and Canadian blueberry exports in 2024 were valued at approximately CAD $695 million, accounting for more than 65% of all Canadian fruit exports. Although lowbush blueberries ( *Vaccinium angustifolium* ) are cultivated throughout Eastern and Atlantic Canada, nearly 94% of Canadian production of highbush blueberries ( *Vaccinium corymbosum* ) occurs in British Columbia, where environmental conditions are highly favourable for the cultivation of this crop (Agriculture and Agri‐Food Canada 2025). These same conditions simultaneously promote the growth and dispersal of bacterial populations on plant surfaces (Donati et al. 2014; Xin et al. 2018; Latchman and Burlakoti 2020; Zhou et al. 2022), however, making Psc a phytopathogen of considerable concern for highbush blueberry production in British Columbia.

In highbush blueberries, Psc infections manifest as blight of leafy tissues or canker of woody tissues, both of which reduce plant health and overall productivity (Hirano and Upper 2000; Lamichhane et al. 2015; Donati et al. 2020). Disease development requires the transition of Psc populations from the epiphytic phase, in which bacteria remain adhered to plant surfaces, to the endophytic phase—which constitutes colonization of internal plant structures and the release of virulence factors that destroy tissues (Hirano and Upper 2000; Kennelly et al. 2007; Melotto et al. 2008; Xin et al. 2018; Donati et al. 2020). Leaf blight is among the most common manifestations of Psc infection in *V. corymbosum*, and can substantially reduce photosynthetic capacity and thus the energy required for fruiting. Environmental conditions common in British Columbia, including cool temperatures, high humidity and frequent precipitation, favour the growth and motility of epiphytic Psc populations and thereby increase the likelihood of endophytic infection (Donati et al. 2014; Xin et al. 2018; de Araujo et al. 2019; Latchman and Burlakoti 2020; Latchman et al. 2026a). Consequently, strategies that aim to reduce Psc populations on plant surfaces before or during the early stages of infection may represent effective means of limiting the establishment and spread of disease on blueberry farms.

Management of Psc infections has traditionally relied on the use of copper‐based biocides to control bacterial burden (McLeod et al. 2017; Lamichhane et al. 2018), but this approach is damaging to the natural environment (Rabiey et al. 2020), can stunt plant growth due to interference with the rhizosphere microbiome (Signorini et al. 2023), and has been waning in efficacy in recent decades due to the spread of plasmid‐borne copper resistance genes (Cooksey 1994; Griffin et al. 2017; Lamichhane et al. 2018). As a result, mitigation is in many cases limited to the costly, unsustainable and sometimes ineffective practice of pruning or outright culling infected plants (DiCarlo 2012; Nakayinga et al. 2021), and new strategies for controlling Psc are therefore urgently required.

One emerging alternative is biocontrol using naturally occurring, highly specialized bactericidal viruses known as (bacterio)phages. Tailed phages are invariably capable of replicating through the lytic cycle, in which rapid viral amplification culminates in lysis of the bacterial host, and phages able to replicate *only* in this manner are termed obligately lytic (Salmond and Fineran 2015; Hobbs and Abedon 2016). In contrast, temperate or lysogeny‐capable (LC) phages can alternatively integrate their genomes into host chromosomes or persist as plasmid‐like elements, permitting passive replication through vertical transmission. LC phages may be suitable in some contexts, but OL phages are generally regarded as safer and more efficacious and therefore remain the gold standard for biocontrol (Skurnik and Strauch 2006; Nale et al. 2016; Al‐Anany et al. 2021; Lauman and Dennis 2023; Cook and Hynes 2025; Fatima et al. 2025; Lauman, Cassell, and Dennis 2026). Since predation by individual phage species is generally restricted to a subset of target strains and readily selects for the emergence of resistant subpopulations, cocktail formulations consisting of multiple distinct bacteriophages are typically employed to simultaneously expand host range, improve antibacterial efficacy and forestall the inevitable development of resistance (Kleinman 2016; Oechslin 2018; Lauman and Dennis 2021).

Phage Agricultural Biocontrol (PhAB), the use of bacteriophages to prevent or control phytopathogenic infections in crops, has been investigated for over a century but has attracted renewed interest in recent decades due to advances in biotechnology and increasing resistance to chemical antimicrobials (Buttimer et al. 2017; Dy et al. 2018; Holtappels et al. 2021). Indeed, successful applications of PhAB have been reported in a wide range of crop‐pathogen systems, with phages variously delivered through inoculations, foliar sprays, soil amendments and irrigation systems to suppress *Burkholderia gladioli* soft rot in onions and mushrooms (Lauman and Dennis 2024), *Burkholderia glumae* seedling rot in rice (Kanaizuka et al. 2023; Supina et al. 2025), *Clavibacter michiganensis* Goss wilt in maize (Kimmelshue et al. 2019), *Dickeya solani* soft rot of potatoes (Carstens et al. 2018), *Erwinia amylovora* fire blight in apples and pears (Schwarczinger et al. 2017), *Ralstonia solanacearum* wilt of tomatoes (Álvarez et al. 2019), *Xanthomonas euvesicatoriae* bacterial spot in peppers (Gašić et al. 2018) and many others (Buttimer et al. 2017; Holtappels et al. 2021), collectively demonstrating the broad applicability of phage‐based biocontrol in agriculture.

Although bacteriophages have been isolated and deployed against Psc lineages infecting several important crops, such as kiwifruit, pear, tomato and leek (Frampton et al. 2014; Rombouts et al. 2016; Flores et al. 2020; Hernandez et al. 2020; Holtappels et al. 2020, 2024; Pinheiro et al. 2020; Skliros et al. 2023), we are aware of no previous work utilizing phages to specifically target blueberry‐tropic strains of this phytopathogen. Since pathovars infecting specific hosts occupy distinct ecological niches and may differ in traits relevant to phage susceptibility, such as surface‐expressed virulence structures doubling as phage receptors, pathovar‐specific phage isolation could be important for the development of effective biocontrol strategies (Oechslin 2018; Xin et al. 2018). In this study, we isolated bacteriophages targeting Psc strains recovered from highbush blueberry plants in southwest British Columbia, and evaluated their suitability for agricultural biocontrol through host‐range analysis, comparative genomics, environmental stability testing, in vitro antibacterial efficacy assays and ex planta validation. Using this pipeline, we constructed phage cocktails capable of longitudinally suppressing Psc colonization of detached *V. corymbosum* leaves, thereby demonstrating the potential of bacteriophages as biocontrol agents against Psc infections of highbush blueberries.

## 2 Methods

### 2.1 Organisms and Media

**FIGURE 1:**
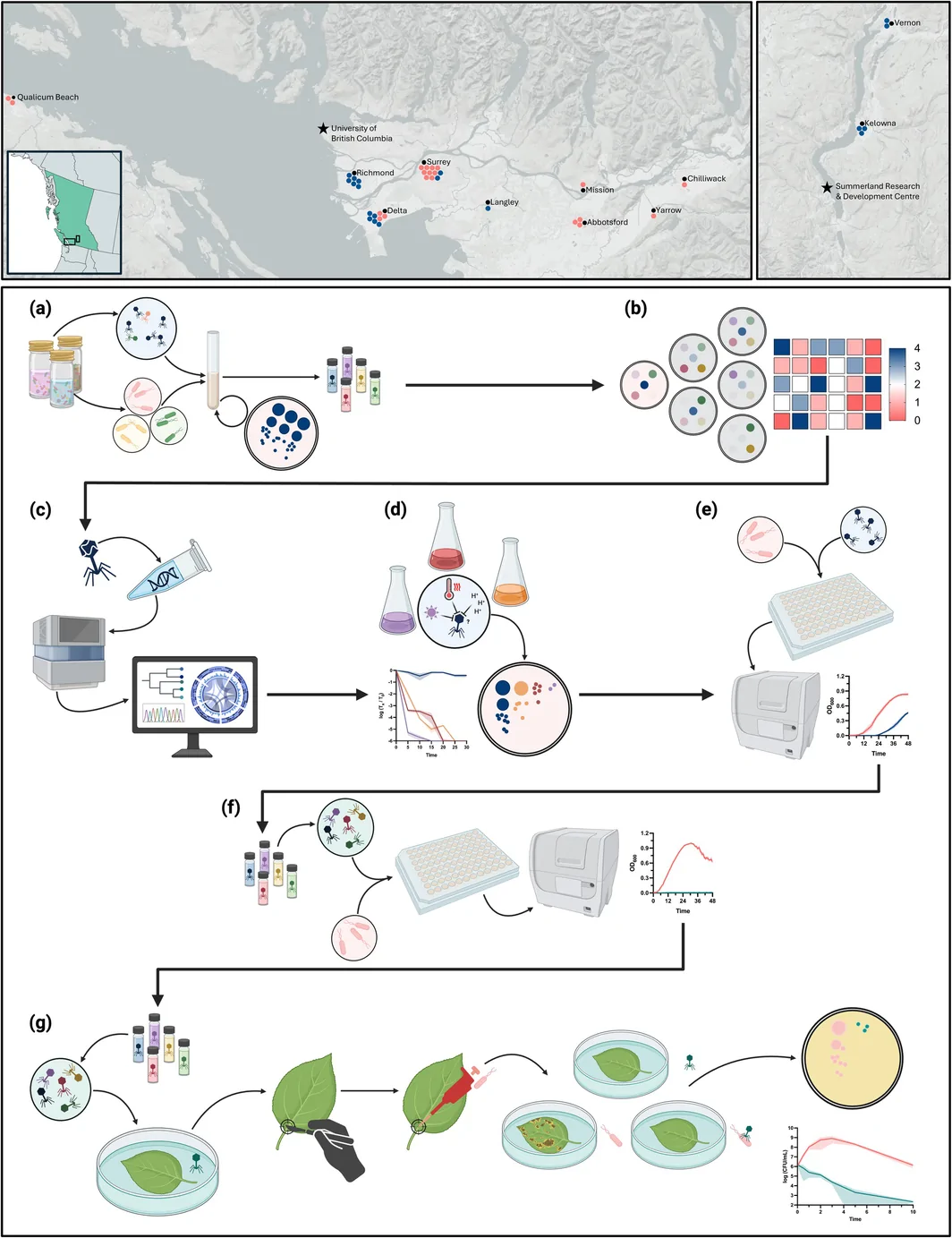
Geographical and experimental framework. Top panels: geographical distribution of sampling sites in and around the lower mainland (left) and Okanagan valley (right) regions of southwestern British Columbia. Names and approximate locations of key research centres (stars) and sampling sites (points) are shown in black, while the number of bacterial and phage isolates from each site is respectively shown using red and blue points. Inset shows British Columbia (green) within the Pacific Northwest region of North America, with the mapped regions highlighted. Bottom panel: graphical representation of experimental workflow. *Pseudomonas syringae* complex strains and phages were isolated and purified (a), and phage host ranges and semiquantitative infection capabilities were assessed to exclude weak specialist phages (b). The genomes of promising biocontrol candidate phages were then sequenced and analysed to characterize taxonomy, identify potentially disqualifying genetic elements, and exclude clonal or near‐clonal duplicates (c). The remaining candidates were then tested to verify stability under simulated environmental conditions (d) and ability to reduce bacterial growth in vitro (e), and cocktails designed to optimize the performance of the most effective individual phages were then formulated and tested in vitro (f). The most effective cocktails were ultimately tested in a prophylactic ex planta highbush blueberry detached‐leaf model of infection, in which decreased bacterial titre over time was used as a measure of antibacterial effect (g).

Twenty strains belonging to the Psc, previously isolated from highbush blueberry plants throughout the lower mainland region of southwestern British Columbia (Latchman et al. 2026a), were used in this study (see Figure 1 and Table S1). Virtually all experiments were performed using Tryptic Soy Broth (TSB) or Tryptic Soy Agar (TSA; BD Difco, Franklin Lakes, NJ), and 20% glycerol‐supplemented TSB was used for the long‐term storage of bacterial stocks at −80°C. Bacteria were incubated statically on TSA plates at 28°C for 48 h, and single colonies were cultured in 5 mL TSB at 28°C, 150 rpm in a gyratory shaker for roughly 18 h before use.

Nineteen *Pseudomonas* phages, variously isolated from sewage samples and Psc‐infected blueberry plants throughout southwestern British Columbia, were used in this study (see Figure 1 and Table S2). Following isolation and purification, phages were stored at 4°C in sodium‐magnesium (SM) buffer (100 mM NaCl, 8 mM MgSO_4_, 50 mM Tris–HCl at pH 7.5, in dH_2_O). All phages were amplified on their respective isolation hosts (see Table S2) using the double agar overlay technique (Adams 1959; Chantapakul et al. 2024), lysate containing amplified phages was resuspended in 10 mL of SM buffer, scrapped off plates and centrifuged at 5000 × *g* for 20 min, and resulting supernatants were sterilized using 0.45 μm MCE filters (Cytiva, Marlborough, MA). Filter‐sterilized lysates were then titred using the double agar overlay technique. Amplifications were repeated to achieve phage titers ≥ 10^9^ PFU mL^−1^, and maintenance of stock titers was routinely verified.

### 2.2 Isolation and Purification of Phages

Phage isolation was conducted using standardized methods described previously (Fong et al. 2017; Chantapakul et al. 2024), with minor modifications. Briefly, sewage samples were centrifuged at 4000 × *g* for 10 min and diluted twofold in double‐strength TSB, while Psc blight samples were added directly to TSB, and all samples were then combined with 1 mL of cultured Psc strains and incubated overnight at 28°C, 150 rpm in a gyratory shaker. Cultured samples were centrifuged at 4000 × *g* for 10 min and sterilized using 0.45 μm MCE filters (Cytiva), and filtrates were subsequently combined with their cognate Psc strains using the double agar overlay technique (Adams 1959; Chantapakul et al. 2024). After 24 h of incubation at 28°C, individual plaques were extracted from agar overlays using micropipette tips and were resuspended in 0.2 mL SM buffer. Suspensions were incubated overnight at room temperature (RT) to permit diffusion of phage particles into the SM buffer, and log‐fold dilutions down to 10^−7^ were created and plated with cognate Psc strains using the double‐agar overlay technique. Plaque resuspension and overlay steps were repeated at least five times or until uniform plaque morphology indicative of a purified phage culture was observed. Final plaque extractions were centrifuged and sterilized twice before being stored in SM buffer at 4°C.

### 2.3 Assessment of Phage Host Range and Exclusion of Weak Specialist Phages

**FIGURE 2:**
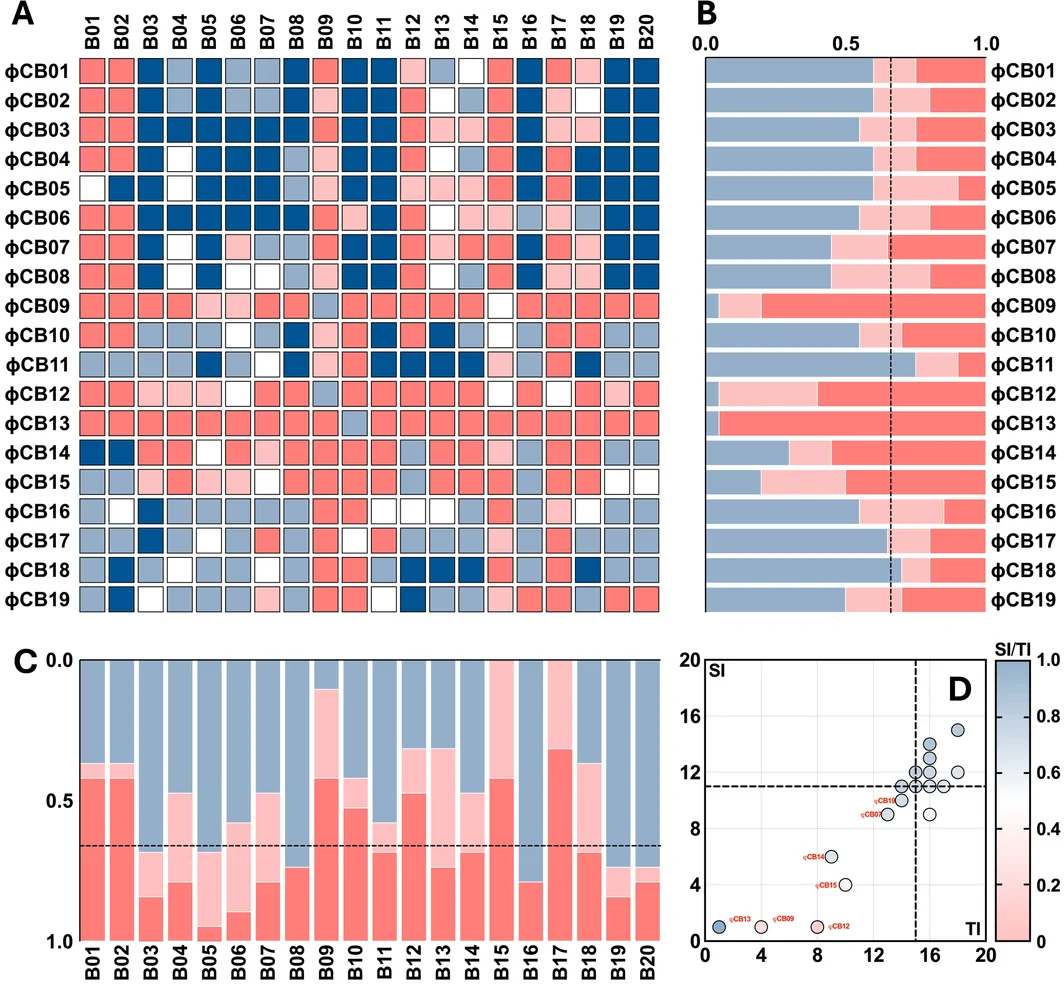
Semiquantitative analysis of *Pseudomonas syringae* complex phage‐host interactions identifies strong generalist phages. Host ranges of *Pseudomonas* phages φCB01–φCB19 targeting *P. syringae* complex strains B01—B20 on solid medium, depicting infection interactions scored as very strong (4; dark blue), strong (3; light blue), moderate (2; white), weak (1; pink) or absent (0; red) based on the visual appearances of clearing zones (A). Proportions of interactions categorized as strong (blue, includes strong and very strong scores), weak (pink, includes moderate and weak scores) or absent (red) presented per phage (B) and per bacterial strain (C), with dashed black lines showing the mean number of infection interactions in each group. Clustering of *Pseudomonas* phages based on numbers of total (TI) and strong interactions (SI), with the proportion of strong interactions (SI/TI) additionally indicated for each phage using a colour gradient (D). Dashed black lines in (D) represent median values for *x*–*y* variables and serve to segregate phages into strong generalists (top right), weak generalists (bottom right), weak specialists (bottom left) and strong specialists (top left). Weak specialist phages (bottom left quadrant, names labelled in red) were excluded from all downstream analyses. All interaction scores depicted herein are based on at least two independent biological replicates.

Host range assessments were performed on all 19 phage isolates using protocols described previously (Chantapakul et al. 2024), with minor modifications. Briefly, 5 μL of 10^9^ PFU mL^−1^ suspensions of each phage were spotted in technical triplicate onto two biologically independent lawns of each of the 20 Psc strains used in this study, and plates were incubated statically at 28°C after air‐drying. Following overnight incubation, zones of inhibition were observed and scored using a semiquantitative scale ranging from 0 to 4, with scores of 4, 3, 2 and 1 respectively representing very strong, strong, moderate and weak levels of bacterial lawn clearance by the phage, and 0 indicating the objective absence of a clearing zone. Scores > 0 were deemed to indicate interactions, while scores ≥ 3 were considered indicative of strong interactions. Numbers of total and strong interactions were determined for each phage, and the median values of these parameters across all 19 phage isolates were used as cut‐off thresholds. Phages that fell below both of these thresholds (*n* = 7) were deemed ‘weak specialists’ and excluded from subsequent analyses (see Figure 2D), while phages that passed at least one of these criteria (*n* = 12) were examined further.

### 2.4 Phage Genome Extraction

Genomic DNA (gDNA) from φCB01, φCB02, φCB03, φCB04, φCB05, φCB06, φCB08 and φCB10 was extracted using the Phage DNA Isolation Kit (Norgen BioTek, Thorold ON), with minor modifications to the manufacturer's protocol. Briefly, 1 mL aliquots of phage lysates titered to ≈10^9^ PFU mL^−1^ were sterilized using 0.45 μm MCE filters (Cytiva), incubated for 15 min at RT with 10 μL of RNase‐free DNase I (Norgen BioTek) to remove host gDNA contamination, and all subsequent steps were performed as specified by the manufacturer.

Bacteriophages φCB11, φCB16, φCB17 and φCB18 consistently produced low gDNA yields when processed using the Phage DNA Isolation Kit, and gDNA from these phages was therefore extracted using an optimized phenol‐chloroform method. Briefly, 0.9 mL aliquots of phage lysates titered to ≈10^9^ PFU mL^−1^ were sterilized using 0.2 μm PES membranes (Thermo Fisher Scientific, Waltham, MA), combined with 0.1 mL of 10× TURBO DNase buffer (Thermo Fisher Scientific), 0.01 mL of TURBO DNase (Thermo Fisher Scientific), and 0.02 mL of RNase A (Qiagen, Germany) in 2.0 mL DNA LoBind tubes (Eppendorf, Germany), and incubated in a thermomixer (Eppendorf) for 30 min at 37°C, 500 rpm. Samples were combined with 0.1 mL of lysis solution (2.5% SDS, 0.25 M EDTA and 0.5 M Tris–HCl buffered to pH 9.0) and incubated for 30 min at 65°C, 500 rpm. Samples were then combined with 0.156 mL of cold 6.4 M KOAc (MilliporeSigma, Burlington, MA) and placed on ice for 30 min before being centrifuged for 10 min at 4°C, 18,000 × *g*. Supernatants were transferred to new tubes, combined with equal volumes of freshly‐mixed 1:1 phenol‐chloroform solution, vortexed, and centrifuged for 10 min at RT, 18,000 × *g*. Upper aqueous phases from phenol‐chloroform washes were transferred to fresh tubes, washed a second time with chloroform alone, and finally combined with 0.4 mL of 20% polyethylene glycol (PEG) 8000 (Thermo Fisher Scientific). Samples were centrifuged for 10 min at 4°C 18,000 × *g*, washed with 0.5 mL of 70% EtOH, and spun again for 5 min at 4°C 18,000 × *g*. gDNA pellets were air dried in a biosafety cabinet and resuspended in 0.05 mL Tris‐EDTA (TE) Buffer.

### 2.5 Whole Genome Sequencing, Assembly and Annotation

The concentration and purity of extracted gDNA was assessed using the NanoDrop 2000 (φCB01–φCB10) and NanoDrop 1000 (φCB11–φCB18) spectrophotometers (Thermo Fisher Scientific), and only gDNA of sufficient purity (A260/A280 ≈1.8; A260/A230 ≈2.0) and concentration (≥ 85 ng μL^−1^) was used for sequencing. For phages φCB01–φCB10, libraries were prepared using the Illumina Nextera XT kit at the UBC Sequencing & Bioinformatics Consortium (University of British Columbia, Vancouver, BC), and sequenced on an Illumina MiSeq platform (Illumina, San Diego, CA) using 2 × 150 bp paired‐end reads. For phages φCB11–φCB18, libraries were prepared using the NxSeq AmpFree Low DNA kit (LGC Biosearch Technologies, Petaluma, CA) at the Molecular Technologies Laboratory (Ottawa Research and Development Center, Agriculture and Agri‐Food Canada, Ottawa, ON), and sequenced on an Illumina MiSeq platform (Illumina) using 2 × 251 bp paired‐end reads.

Raw reads were quality‐filtered and adapter‐trimmed using fastp v0.24.1 (Chen et al. 2018; Chen 2023), and phage genomes were *de novo* assembled using SPAdes v3.14.2 (Bankevich et al. 2012). For φCB01 and φCB10, reads were subsampled prior to assembly to improve assembly contiguity. All assembly outputs were screened for putative phage‐derived contigs based on contig length, sequencing coverage, and similarity to known phage genomes, and dominant phage‐associated contigs were selected as representative genomes for downstream analyses. Genome completeness and terminal structure were assessed using CheckV v1.0.1 (Nayfach et al. 2021). Phage genomes were annotated using Pharokka v1.9.1 (Bouras et al. 2023), and predicted coding sequences (CDSs) were used for downstream functional screening. All software was run under default parameters. All sequenced phage genomes have been deposited as part of an NCBI BioProject, with accession number PRJNA1477681.

### 2.6 Bioinformatic Analysis

Putative taxonomic affiliations and reference genomes were identified using ICTV TaxaBLAST and comparison with the ICTV taxonomy database (Black et al. 2026). Whole‐genome phylogenetic relationships were inferred using VICTOR based on Genome‐BLAST Distance Phylogeny (GBDP) distances calculated using the *D*_0_ distance formula and the FASTME tree‐building method (Meier‐Kolthoff and Göker 2017), and pairwise intergenomic similarities were calculated for all phages using VIRIDIC (Moraru et al. 2020). When necessary, phage genomes were reoriented to begin at conserved packaging or structural genes, and comparative genome organization was visualized using clinker v0.0.32 (Gilchrist and Chooi 2021).

Phage lifestyle prediction was conducted using BACPHLIP (Hockenberry and Wilke 2021), and predicted CDSs were manually screened for canonical lysogeny‐associated genes, including integrases, excisionases, repressors and recombinases. Phages were also screened for potential antimicrobial resistance genes using AMRFinderPlus v4.2.7 (Feldgarden et al. 2021) with the NCBI reference database (release 2026‐03‐24.1), and for metal and biocide resistance genes using ABRicate v1.4.0 with the BacMet2 database (Pal et al. 2014). Putative phage morphology was inferred from predicted structural gene content, including genes encoding capsid, packaging, tail, baseplate and receptor‐binding proteins.

### 2.7 Stability Assays

Phage isolates were assessed for viability following exposure to radiation, freezing, heat and pH stresses. In radiation stress tests, phages were diluted to 10^7^ PFU mL^−1^ in SM buffer and aliquoted into a 96‐well plate positioned ≈30 cm from a hand‐held UV lamp (AnalytikJena, Germany) radiating at 365 nm (UV‐A, relevant to field conditions) or 254 nm (UV‐C, relevant to food disinfection protocols) for up to 60 min, and viable phage concentrations were quantified using the double‐agar overlay technique (Adams 1959; Chantapakul et al. 2024) on log‐fold diluted samples collected after 0, 15, 30, 45 and 60 min of exposure. In temperature and pH stress tests, phages were diluted to 10^7^ PFU mL^−1^ and aliquoted into 1.7 mL tubes (Eppendorf), but temperature‐challenge samples were diluted in SM buffer and stored at −20°C, 4°C, 22°C or 37°C while pH‐challenge samples were diluted and stored in modified SM buffers titered to pH 4, 6 or 8. Samples were stored for up to 30 days, and viable phage titres were quantified using the double‐agar overlay technique on log‐fold diluted samples collected after 0, 1, 3, 5, 7, 10, 15, 20, 25 and 30 days of exposure. Data are expressed as the means of log[*T*_x_/*T*_0_] and log[*T*_0_/*T*_x_] values in Figure 4 and Table 2, respectively, where *T*_x_ and *T*_0_ respectively represent phage concentrations at indicated and initial timepoints.

### 2.8 Planktonic Killing Assays and Cocktail Assembly

Planktonic Killing (PK) assays were performed and analysed as described previously (Lauman and Dennis 2023), with minor modifications. Eight Psc strains (see Table S1) were selected based on their virulence in *V. corymbosum* (Latchman et al. 2026b). Overnight cultures of these strains were diluted to 2 × 10^6^ CFU mL^−1^ in fresh TSB, and 0.05 mL of diluted cultures were combined with equivalent volumes of SM buffer (untreated growth controls) or phage stocks diluted to 2 × 10^8^ PFU mL^−1^ in SM buffer, in wells in a flat‐bottomed 96‐well plate (Corning, Corning, NY), establishing infections at initial MOIs of 100. Negative controls containing sterile TSB and SM buffer were included to confirm sterility throughout the experimental period. Plates were incubated in a SpectraMax M2/M2e microplate spectrophotometer (Molecular Devices, Sunnyvale, CA) at 28°C for 48 h, with orbital shaking prior to each reading, and OD_600_ readings were collected automatically every 30 min.

Output data were analysed using the Growth Reduction Coefficient (GRC) approach described previously (Lauman and Dennis 2023). Briefly, the area ($A_{x}$) under the OD_600_ versus time curve for treatment with phage x was computed using the trapezoid approximation method:

$$A_{x}=\int_{t_{i}}^{t_{f}}{\mathrm{OD}}_{600}\left(x\right)dt$$

and outputs were mathematically transformed through comparison with the area ($A_{0}$) under the OD_600_ versus time curve for the *untreated* strain to yield the GRC for phage x on that strain:

$${\mathrm{GRC}}_{x}=1-\frac{A_{x}}{A_{0}}$$

Individual phages were ranked based on their median GRCs across all eight Psc target strains (see left side of Figure 5G) to identify the most and least effective phages. To determine whether the growth suppression capacity of the most effective phages could be improved through combination with phages that are less effective on their own, the two most effective phages were variously combined with the four least effective phages to construct eight unique phage cocktails (φC), designated φC_A‐H, containing between three and five phage variants (see Table S3 for details). φCs were subsequently tested against all eight Psc target strains using PK assays, following the procedure described above for individual phages, and the four most effective φCs (see right side of Figure 5G) were selected for downstream trials ex planta.

### 2.9 Detached Leaf Assays

Young (≤ 2 weeks old) leaves were picked from highbush blueberry plants of the Draper variety and were used to evaluate the antibacterial efficacy of *Pseudomonas* phage cocktails ex planta. Leaves were removed from plants no earlier than 24 h prior to inoculation, randomly assigned to treatment groups, and surface sterilized with 0.5% NaClO before being rinsed thrice in sterile distilled water. Leaves were submerged in 10 mL of SM buffer or 10^10^ PFU mL^−1^ phage cocktail solutions for 1 min and were subsequently left to dry in sterile petri plates for 2 h. Incisions were then made at the bases of leaves and 0.025 mL of TSB or 10^8^ CFU mL^−1^ subcultures of Psc strains B04 or B11 were inoculated into the wounds. Leaves were left in unsealed Petri plates for 2 h to permit bacterial absorption into wounds, and the plates were subsequently parafilm‐sealed to create humidity chambers and were transferred to a 10°C growth chamber with 12‐h light–dark cycles for up to 10 days of incubation.

Samples were collected at 0, 0.5, 1, 2, 3, 5 and 10 days post‐infection, and were used to estimate both bacterial and phage concentrations in leaves. At each timepoint, individual leaves were inserted into 1.7 mL tubes (Eppendorf) containing 1 mL of SM buffer, and were incubated in an orbital shaker at 28°C, 150 rpm for 30 min to dislodge bacteria and phage particles into solution. 0.5 mL aliquots of the collected samples were sterilized using 0.45 μm MCE filters (Cytiva) to remove bacteria or other large debris, and log‐fold dilutions of the filtrate were then used to quantify phage concentration using the double agar overlay method (Adams 1959; Chantapakul et al. 2024). Remaining 0.5 mL aliquots were centrifuged at 4000 × *g* for 10 min to pellet cells, which were resuspended and diluted in TSB and plated on TSA plates to quantify concentrations of Psc strains. Following enumeration, growths on TSA plates were restruck onto King B agar plates (MilliporeSigma) for 24 h of static incubation at 28°C, and were inspected under a blacklight to confirm, via fluorescence, that observed colonies belonged to the Psc. Bacterial quantification was also conducted for leaves inoculated only with TSB, and lack of detectable bacterial growth in these samples was used to confirm the efficacy of sterilization and general validity of results. In cases where bacterial or phage density fell below the limit of detection (LOD; 200 CFU mL^−1^), values of LOD/2 were assigned to facilitate statistical analysis.

To evaluate treatment efficacy across the entire experimental period and permit straightforward comparisons between the overall ex planta antibacterial efficacies of individual cocktails within and between target strains, as well as with data obtained in vitro, we used a variant of the established GRC metric (Lauman and Dennis 2023), the ex planta GRC, which is calculated identically (see previous section) but uses log(CFU mL^−1^) as a computation parameter:

$$A_{x}=\int_{t_{i}}^{t_{f}}\log\left(\mathrm{CFU}\;{\mathrm{mL}}^{-1}\right)\left(x\right)dt$$

Three independent biological replicates were included for each treatment at each time point, consistent with the exploratory nature of the ex planta evaluation. Given the small sample size and the discrete distribution of the bacterial density data, including several observations below the LOD, differences among treatment groups were evaluated using the Kruskal–Wallis test followed by Dunn's multiple comparisons test.

### 2.10 Statistical Analysis

Details of individual statistical analyses are provided in figure legends and, where necessary, in experiment‐specific methods sections. All descriptive statistics, linear regressions, and ANOVAs and post hoc tests were conducted in GraphPad Prism v11.0.0 (GraphPad Software, San Diego, CA). In all analyses, *p* < 0.05 was considered the threshold of statistical significance. Figures were created using GraphPad Prism v11.0.0 (Graphpad Software) and BioRender (BioRender, Toronto, ON).

## 3 Results

### 3.1 Strong Generalist Phages Targeting the *P. syringae* Complex Are Environmentally Abundant

To ensure that isolated phages target strains relevant to pathogenesis in highbush blueberries, we utilized a panel of 20 Psc strains previously collected from several varieties of diseased *V. corymbosum* plants from seven locations in and around the lower mainland region of British Columbia (Figure 1 and Table S1; see Latchman et al. 2026a). Wastewater and *V. corymbosum* blight samples from six sites in the lower mainland and Okanagan valley regions were subsequently collected and incubated with our Psc isolates, and ultimately yielded 19 relatively stable bacteriophage isolates (Figure 1 and Table S2).

Using a semiquantitative spotting analysis of host range, we found that roughly two‐thirds (66.2%) of all tested phage‐bacterium interactions resulted in observable evidence of infection (i.e., zones of clearing; Figure 2A). Individual host ranges were highly variable, however, with isolate φCB13 infecting only a single strain while phages φCB05 and φCB11, on the opposite end of the spectrum, infected 90% of our strain panel (Figure 2B). Notably, the median number of total infection interactions (i.e., viable hosts) per phage was 75% of our host panel, comparable to at least one recent analysis of *P. syringae* phages (Rombouts et al. 2016) but substantially higher than values generally reported for phages infecting the related ESKAPE pathogen *Pseudomonas aeruginosa* (Ceyssens et al. 2011; Forti et al. 2018; Kauppinen et al. 2021; Dai et al. 2023). Strong infection interactions (shades of blue) were observed more frequently than weak interactions in 84% of our phage isolates and collectively accounted for nearly 70% of all infection interactions (Figure 2B), suggesting that in general, our phages are capable of infecting a wide range of agriculturally relevant Psc isolates. No meaningful correlation was found between host range size and the proportion of interactions that were categorized as strong (Figure S1A), however, meaning that a broad host range does not imply strong overall infectivity and both variables must therefore be optimized independently.

Sensitivity to phages varied considerably among individual Psc strains, with hypersensitive isolates B05 and B06 respectively infected by 94.7% and 89.5% of our phages, while strains B17 and B15 exhibited weak sensitivities to only 31.6% and 42.1% of our phage isolates, respectively (Figure 2C). Excluding B17 and B15, all Psc isolates were strongly infected by at least two phages, providing further support for our ability to control blueberry‐associated Psc lineages with the phages isolated herein. Curiously, we found a modest but significant (*r* = 0.49; *p* = 0.03) correlation between the number of phages to which a strain is sensitive and the proportion of such infections that are strong (Figure S1B), suggesting that the size of a strain's phage susceptibility range could be predictive of average sensitivity to those phages.

Since optimization of both the host ranges (total number of interactions) and infection efficacies (number of strong interactions) of phages is critical for applications in biocontrol, we utilized the median values for these parameters as viability thresholds, and phages that fell below *both* of these thresholds (φCB07, φCB09, φCB12, φCB13, φCB14, φCB15 and φCB19) were identified as weak specialists and excluded from further analyses (Figure 2D). Isolates that passed both thresholds (φCB01, φCB02, φCB03, φCB04, φCB05, φCB06, φCB11, φCB16, φCB17, φCB18) were designated ‘ideal’ strong generalists, while φCB08 and φCB10 are respectively singular examples of weak‐generalists and strong specialists. Phages belonging to the latter three categories were deemed appropriate candidates for agricultural biocontrol and were further investigated in this study.

### 3.2 *Pseudomonas* Biocontrol Candidate Phages Exhibit Extensive Phylogenomic Diversity

**FIGURE 3:**
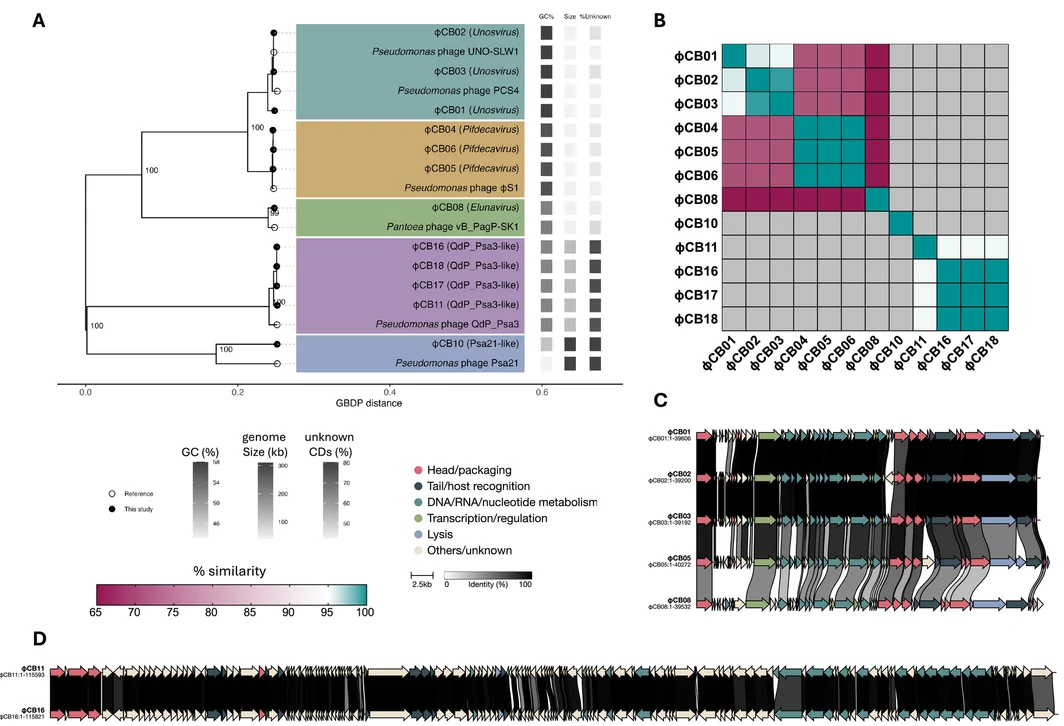
*Pseudomonas* biocontrol candidate phages exhibit extensive phylogenomic diversity. (A) Whole‐genome phylogeny of 12 biocontrol candidate phage isolates (black nodes) and their closest known relatives (white nodes) reconstructed using the VICTOR pipeline. Evolutionary relationships were inferred from Genome‐BLAST Distance Phylogeny (GBDP) whole‐genome distances. Node values correspond to GBDP pseudo‐bootstrap support values, while branch lengths are proportional to whole‐genome distances inferred by GBDP. Adjacent heatmaps summarize GC content (%), genome size (kb), and the proportion of predicted CDSs assigned as hypothetical proteins (%) for each phage genome, with darker shades indicating higher values. (B) Intergenomic similarity matrix of the 12 phage isolates generated using VIRIDIC. Colour intensity corresponds to pairwise intergenomic similarity (%), with shades of teal and light maroon denoting values above and below the 95% species‐demarcation threshold (white), respectively, while pairwise similarities < 65% and < 1% are shown in dark maroon and grey, respectively. (C, D) Comparative genomic analyses of the multi‐member phage clusters identified in Panels (A) and (B), including Autotranscriptaviridae cluster 1 (φCB01, φCB02, φCB03, φCB05 and φCB08; C) and QdP_Psa3‐like cluster 2 (φCB11 and φCB16; D). Genes are represented by arrows coloured according to predicted function, and homologous proteins identified by Clinker are connected by shaded links, with shading intensity corresponding to amino acid sequence similarity.

Taxonomic classification based on whole‐genome sequencing of our 12 candidate phages revealed five species‐level lineages, several of which were represented by multiple clonal duplicates and both similar and highly diverged variants (Table 1, Figure 3A,B). φCB01, φCB02 and φCB03 are variants of the same species belonging to the genus *Unosvirus*, within which φCB02 and φCB03 are highly similar (99.4% intergenomic similarity) while φCB01 is highly diverged from both φCB02 (95.9%) and φCB03 (95.4%). Interestingly, φCB01 and φCB02 are most closely related to *Pseudomonas Unosvirus* PCS4 (93.1% and 96.1%, respectively), while φCB03 is related to *Pseudomonas Unosvirus* UNO‐SLW1 (98.2%), suggesting that these three isolates may be divergent variants of existing *Unosvirus* phages. φCB04, φCB05 and φCB06 are clonal duplicates (100% intergenomic similarity) belonging to the genus *Pifdecavirus*, and are most closely related to *Pseudomonas* phage φS1 (96.7%). Intriguingly, φCB01 and φCB06 were isolated from wastewater samples extracted in Langley and Delta, respectively, while φCB02–φCB05 were found in samples from Richmond, suggesting that these two lineages may have broad distribution throughout the lower mainland (see Figure 1 and Table S2). φCB08 may be a novel species belonging to the genus *Elunavirus*, and is most closely related to *Pantoea* phage vB_PagP‐SK1 (89.1%). φCB01–φCB06 and φCB08 belong to the family *Autotranscriptaviridae*, have relatively small (≈40 kb) genomes encoding ≈55 CDSs, and form a cluster in which the *Unosvirus* variants are somewhat related to the *Pifdecavirus* clones (≈68.3%) while both genera are distantly related to *Elunavirus* φCB08 (≈22%). φCB16, φCB17 and φCB18 are clonal duplicates belonging to the same species as φCB11, from which they are highly diverged (≈95.3%). These phages may collectively represent a novel species within an as yet undefined genus, and are most closely related to *Pseudomonas* phage QdP_Psa3, which is related more closely to the φCB16/CB17/CB18 clones (91.9%) than to φCB11 (91.2%). Unlike the *Autotranscriptaviridae*, to which they have no meaningful relatedness, the phages forming this second cluster have relatively large genomes (≈116 kb) encoding roughly 170 CDSs. Notably, φCB16–φCB18 were recovered from *V. corymbosum* blight samples collected in Delta while φCB11 was found in a wastewater sample from Kelowna, nearly 300 km away, potentially suggesting a broad geographic distribution for this lineage throughout southwestern British Columbia (see Figure 1 and Table S2). φCB10, a ≈310 kb jumbo phage with 476 CDSs, is unrelated to our other lineages, is most closely related to *Pseudomonas* jumbo phage Psa21 (53.2%), and may be the founding member of a novel genus. In line with these findings, the proportion of predicted CDSs assigned as hypothetical proteins is nearly twice as high in φCB10 and the QdP_Psa3‐like phages relative to those in the *Autotranscriptaviridae* cluster, implying that these larger phages belong to relatively undersampled or previously unknown lineages (Table 1, Figure 3A,B).

**TABLE 1:** Genomic and taxonomic characteristics of *Pseudomonas* phages.

Phage isolate	Genome size	GC content	Total CDSs	CDSs of unknown function (%)	Family	Genus	Closest relative (% similarity)	Predicted lifestyle (probability)	Predicted morphology
φCB01	39,606 bp	58%	55	24 (43.6%)	*Autotranscriptaviridae*	*Unosvirus*	PCS4 (93.1%)	OL (0.988)	Podovirus
φCB02	39,200 bp	58%	55	25 (45.5%)	*Autotranscriptaviridae*	*Unosvirus*	PCS4 (96.1%)	OL (0.998)	Podovirus
φCB03	39,192 bp	58%	57	26 (45.6%)	*Autotranscriptaviridae*	*Unosvirus*	UNO‐SLW1 (98.2%)	OL (0.998)	Podovirus
φCB04	40,272 bp	56%	56	25 (44.6%)	*Autotranscriptaviridae*	*Pifdecavirus*	φS1 (96.7%)	OL (0.999)	Podovirus
φCB05	40,272 bp	56%	56	25 (44.6%)	*Autotranscriptaviridae*	*Pifdecavirus*	φS1 (96.7%)	OL (0.999)	Podovirus
φCB06	40,272 bp	56%	56	25 (44.6%)	*Autotranscriptaviridae*	*Pifdecavirus*	φS1 (96.7%)	OL (0.999)	Podovirus
φCB08	39,532 bp	52%	52	23 (44.2%)	*Autotranscriptaviridae*	*Elunavirus*	vB_PagP‐SK1 (89.1%)	OL (1.000)	Podovirus
φCB10	310,574 bp	47%	476	382 (80.3%)	Not defined		Psa21 (53.2%)	OL (0.800)	Myovirus
φCB11	115,593 bp	52%	168	128 (76.2%)	Not defined		QdP_Psa3 (91.2%)	OL (0.688)	Myovirus
φCB16	115,821 bp	52%	170	131 (77.1%)	Not defined		QdP_Psa3 (91.9%)	OL (0.688)	Myovirus
φCB17	115,821 bp	52%	170	131 (77.1%)	Not defined		QdP_Psa3 (91.9%)	OL (0.688)	Myovirus
φCB18	115,693 bp	52%	170	131 (77.1%)	Not defined		QdP_Psa3 (91.9%)	OL (0.688)	Myovirus

All seven isolates belonging to the *Autotranscriptaviridae* were predicted to be obligately lytic (OL; probability ≥ 0.988) by BACPHLIP, while the QdP_Psa3‐like phages yielded an intermediate score of 0.688 (Table 1). Although a virulent lifestyle was also predicted for jumbo phage φCB10 (0.800), this finding should be interpreted with caution since jumbo phages are underrepresented in the BACPHLIP training set. Consistent with these predictions, preliminary genome annotations did not identify integrases, excisionases or other canonical lysogeny‐associated genes (Table S4). Moreover, no antimicrobial or metal resistance genes were identified in the genomes of these phages, supporting their potential suitability as biocontrol agents. Based on structural gene content, *Autotranscriptaviridae* isolates φCB01–φCB06 and φCB08 are predicted to have podovirus morphologies while φCB10, φCB11 and φCB16–φCB18 are thought to be myoviruses (Table 1 and Table S5), but confirmation via electron micrography will be required for definitive characterization.

In all subsequent genomic and functional analyses, φCB05 and φCB16 were selected as the representatives of clonal groups φCB04–φCB06 and φCB16–φCB18, respectively, since they have the widest host ranges within their groups. Comparative genomic analyses of *Autotranscriptaviridae* Cluster 1 (Figure 3C) and QdP_Psa3‐like cluster 2 (Figure 3D) show that the majority of within‐species differences are associated with genes of unknown function, while differences between genera are predictably distributed throughout entire genomes. Interestingly, the GC content of jumbo phage φCB10 is substantially lower than values seen in our other *Pseudomonas* phage lineages (Table 1), possibly suggesting that this phage may be incompletely adapted to infecting *Pseudomonas* species.

### 3.3 *Pseudomonas* Phages Exhibit Heterogeneous Environmental Stability

**FIGURE 4:**
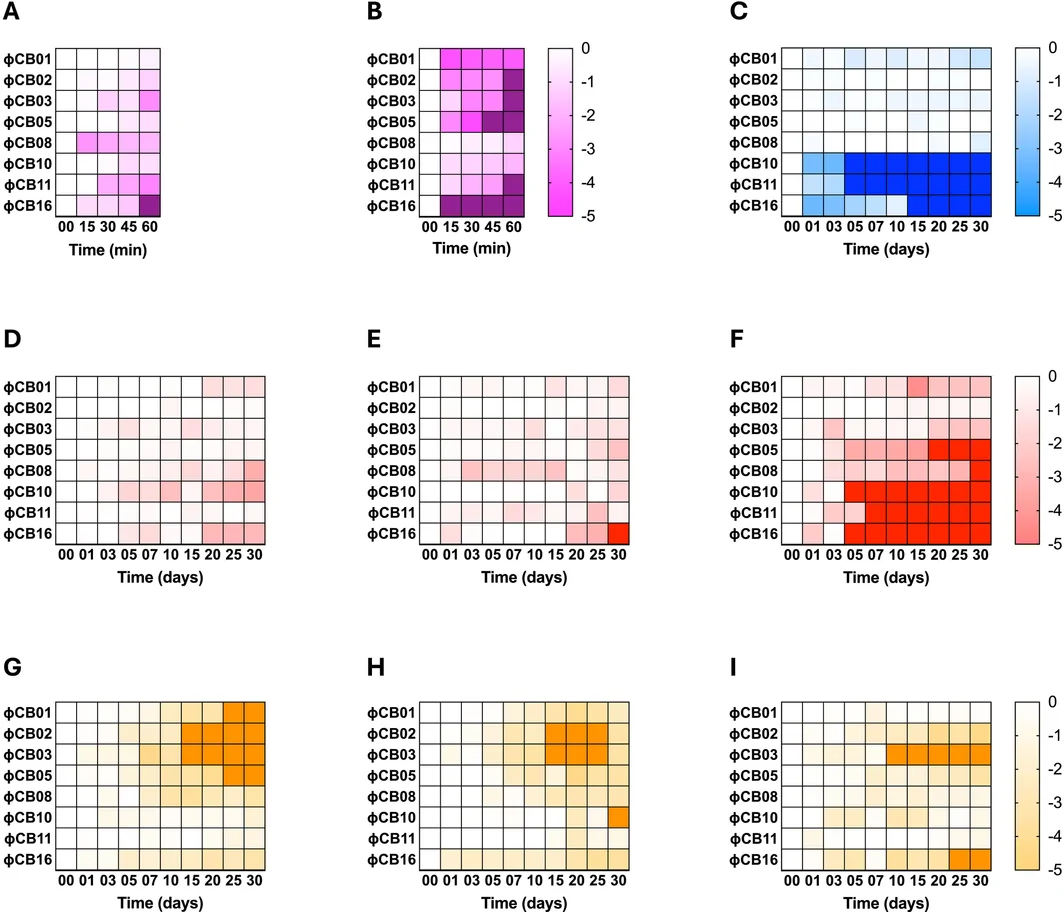
*Pseudomonas* phages exhibit heterogenous environmental stability. Effects of radiation (violet) at 365 nm (UVA; A) and 254 nm (UVC, B), freezing (blue) at −20°C (C), heating (red) at 4°C (D), 22°C (E) and 37°C (F) and pH stress (orange) at pH 4.0 (G), 6.0 (H) and 8.0 (I) on the viability of Pseudomonas phages over time. Data are presented as log‐transformed proportions of starting titers remaining viable at each timepoint, with white representing no loss and increasingly darker shades depicting titre losses at increasing orders of magnitude, while the darkest shades indicate titre losses to levels below the limit of detection (log [*T*_x_/*T*_0_] < −5). Note that radiation data were collected over the course of an hour, while all other data were obtained over a month. All presented values depict the means of three independent biological replicates.

To determine the extent to which our phage isolates tolerate environmental conditions to which they might be exposed in agricultural applications, we next conducted stability assays in which the titres of phages variously exposed to ultraviolet radiation, freezing, heat and pH stress were monitored over time (Figure 4, key timepoints in Table 2). The destabilizing effects of radiation, monitored over the course of a single hour, were substantial but predictably differed considerably based on the wavelength of the light. After 60 min of exposure to UV‐A at 365 nm, only φCB16 exhibited a titre drop to below the limit of detection (*p* = 0.001) while all other phages exhibited significant (*p* ≤ 0.0375) reductions in viable titre, ranging between 0.4 and 3.0 orders of magnitude, but nevertheless persisted at detectable levels (Figure 4A). In contrast, exposure to UV‐C at 254 nm triggered 1.2, 1.9, and 4.2 log‐fold decreases in the viable titers of φCB08 (*p* = 0.0105), φCB10 (*p* = 0.0073) and φCB01 (*p* = 0.0056), respectively, while the titers of all our other phages fell below the limit of detection (all *p* = 0.0066; Figure 4B and Table 2).

**TABLE 2:** Environmental stability of *Pseudomonas* phages.

Phage	Stability^a^															
	Radiation (nm)		Temperature (°C)								pH					
	365 (1 h)	254 (1 h)	−20 (1 day)	−20 (30 day)	4 (1 day)	4 (30 day)	22 (1 day)	22 (30 day)	37 (1 day)	37 (30 day)	4 (1 day)	4 (30 day)	6 (1 day)	6 (30 day)	8 (1 day)	8 (30 day)
φCB01	0.4	4.1	0.4	1.4	0.0	1.4	0.0	0.0	0.0	2.5	0.0	BDL	0.0	2.5	0.0	0.0
φCB02	1.2	BDL	0.0	0.0	0.0	0.0	0.0	0.0	0.0	0.4	0.0	BDL	0.0	3.5	0.0	4.5
φCB03	2.9	BDL	0.0	0.5	0.0	0.0	0.0	0.0	0.0	2.4	0.0	BDL	0.0	3.5	1.0	BDL
φCB05	0.9	BDL	0.0	0.0	0.0	0.0	0.0	2.5	0.0	BDL	0.0	BDL	0.0	3.5	0.0	3.5
φCB08	1.9	1.2	0.0	0.8	0.0	3.3	0.0	0.0	0.0	BDL	0.0	3.4	0.0	3.1	0.0	1.1
φCB10	0.9	1.9	3.2	BDL	0.0	3.5	0.0	0.0	1.3	BDL	0.0	1.7	0.0	BDL	0.0	0.9
φCB11	3.0	BDL	1.6	BDL	0.0	0.0	0.0	0.0	0.0	BDL	0.0	1.3	0.0	1.0	1.0	0.9
φCB16	BDL	BDL	3.4	BDL	0.0	2.8	1.4	BDL	2.1	BDL	0.0	3.3	0.0	3.6	0.0	BDL

^a^ Data are expressed as mean log‐fold decreases in titre, log[*T*_0_/*T*_x_], where *T*_x_ and *T*_0_ are mean phage concentrations at the indicated timepoint and start time, respectively. Zeroes indicate statistically insignificant changes in titre at the relevant timepoint, while BDL (below detection limit) denotes cases where the titre dropped below the limit of detection.

The impact of freezing, heat and pH stresses on viable phage titers were monitored across a period of 30 days, and varied substantially between phages both within and between stress group. The effects of freezing at −20°C (Figure 4C) were particularly polarizing, with phages separating into two groups based on the magnitude of their susceptibilities. Viable titers of predicted myoviruses φCB10, φCB11 and φCB16 dropped by 3.2, 1.6 and 3.4 order of magnitude, respectively, after a single day of exposure (*p* ≤ 0.0061), and fell below the limit of detection after 30 days (all *p* = 0.0026). Conversely, of the five predicted podovirus isolates only φCB01 exhibited a statistically significant (*p* = 0.0351) but minor 0.4 log‐fold drop in titre after 1 day, and after 30 days the viable titers of φCB01, φCB03 and φCB08 were reduced by 1.4 (*p* = 0.0036), 0.5 (*p* = 0.0415), and 0.8 (*p* = 0.011) orders of magnitude, respectively. Interestingly, φCB02 and φCB05 did not exhibit significant losses in viable titers over the 1 month period (Figure 4C, Table 2). Collectively, these data suggest that podovirus capsids may have adaptations which permit greater stability at freezing temperatures.

Significant destabilization due to heat was seen only sparingly after 1 day of exposure, with no significant differences observed at 4°C (Figure 4D), only φCB16 showing a significant (*p* = 0.0491) 1.4 log‐fold reduction at 22°C (Figure 4E), and only φCB10 and φCB16 titres dropping by 1.3 (*p* = 0.0483) and 2.1 (*p* = 0.0395) orders of magnitude at 37°C (Figure 4F). The effects of heat were much more pronounced, however, after 30 days of exposure, with certain phages consistently exhibiting compromised stability. At 4°C (Figure 4D), for instance, φCB01 only exhibited a modest 1.4 log‐fold titre loss (*p* = 0.0037), but φCB08, φCB10 and φCB16 titres dropped 2.8–3.5 log‐fold (all *p* = 0.0027). φCB16 titre also fell below the limit of detection (*p* = 0.0473) at 22°C (Figure 4E), while the titre of CB05 dropped by 2.5 (*p* = 0.048) orders of magnitude and the viability of all other phages remained constant. Indeed, certain isolates appeared to be more stable at 22°C than at the typical 4°C storage temperature, which raises questions about the universality of phage storage conventions. After 30 days of storage at 37°C (Figure 4F), the titres of all three myoviruses as well as podoviruses φCB05 and φCB08 fell to undetectable levels (all *p* = 0.0004) while those of φCB01 and φCB03 respectively dropped by 2.5 and 2.4 orders of magnitude (*p* = 0.0004), and the viable titre of φCB02 fell by only 0.4 log‐fold (*p* = 0.0199). Curiously, φCB02 exhibits the lowest overall sensitivity to the effects of heating, in addition to its resistance to freezing (Figure 4C, Table 2), possibly suggesting capsid thermostability adaptations in this phage.

The effects of pH on phage stability were also minimal after 1 day of exposure, particularly at acidic conditions (pH 4 and 6) under which no significant differences were observed (Figure 4G,H). At pH 8 (Figure 4I) only φCB03 and φCB11 exhibited modest 1.0‐log titre drops (*p* ≤ 0.0053) after 1 day, while all other isolates remained stable. After 30 days of exposure, high acidity had the strongest impact on viability, with titers of φCB01, φCB02, φCB03 and φCB05 all falling below the detection limit (all *p* = 0.0007) while φCB08, φCB10, φCB11 and φCB16 titers dropped by 1.7–3.4 orders of magnitude (*p* ≤ 0.0012; Figure 4G). The effects of low acidity were less pronounced (Figure 4H), with only φCB10 titre dropping below the limit of detection (*p* = 0.0221), but other phages nevertheless exhibited titre drops ranging between 1.0 and 3.5 orders of magnitude (*p* ≤ 0.0363). Similarly, 30‐day exposure to low‐level alkalinity destabilized φCB03 and φCB16 to levels below the detection limit (all *p* = 0.0073) and reduced the titers of φCB02, φCB05, φCB08, φCB10 and φCB11 by 0.9–4.5 orders of magnitude (*p* ≤ 0.0169), but had no discernable impact on the viability of φCB01 (Figure 4I).

Despite substantial heterogeneity in individual stability profiles, all tested candidate phages remained viable under at least a subset of agriculturally relevant environmental conditions, supporting their potential application as agricultural biocontrol agents.

### 3.4 *Pseudomonas* Phages Control the Planktonic Growth of *P. syringae* Complex Species

**FIGURE 5:**
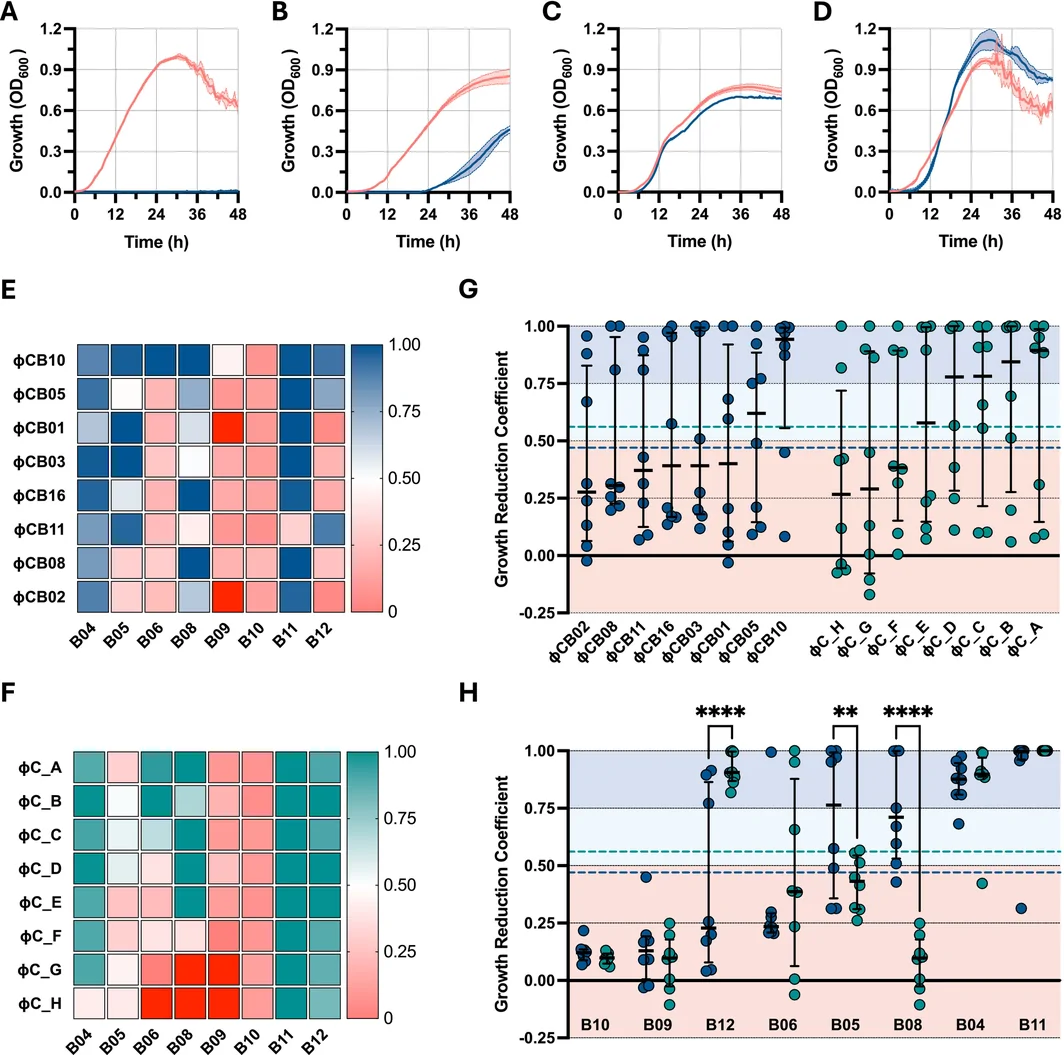
Phages control planktonic growth of *Pseudomonas syringae* complex species. Bacterial growth curves representative of cases where growth is (A) abolished, (B) temporarily suppressed, (C) unaffected or (D) improved by phage treatment (blue) relative to untreated controls (pink). Growth Reduction Coefficients (GRCs) for *P. syringae* complex strains treated with single phages (dark blue) or phage cocktails (teal), presented as heatmaps detailing individual interactions (E, F), and as dot plots depicting GRCs per treatment (G) and per strain (H), organized by median treatment effectiveness (top to bottom in E, F; right to left in G, H). All presented datapoints depict the means of at least three independent biological replicates, for which SD is shown as shaded regions in Panels (A–D), while the central bars and error bars in Panels (G, H) show the median and IQR for each group. Positive GRCs are shown using blue‐pink and teal‐pink gradients in Panels (E) and (F) respectively while negative values are depicted in bright red. Dark blue and teal dashed lines in Panels (G) and (H) respectively show the median GRCs of individual and cocktail treatments. Regions shaded dark blue (GRC > 0.75), light blue (0.75 ≥ GRC ≥ 0.50) and pink (GRC < 0.50) in Panels (G, H) correspond to GRC ranges deemed strong, acceptable or poor, respectively. Differences between groups in (H) were identified using two‐way ANOVAs followed by Fisher's LSD tests, with **** and ** indicating *p* < 0.0001and *p* < 0.01, respectively. For simplicity, only significant differences are shown.

**FIGURE 6:**
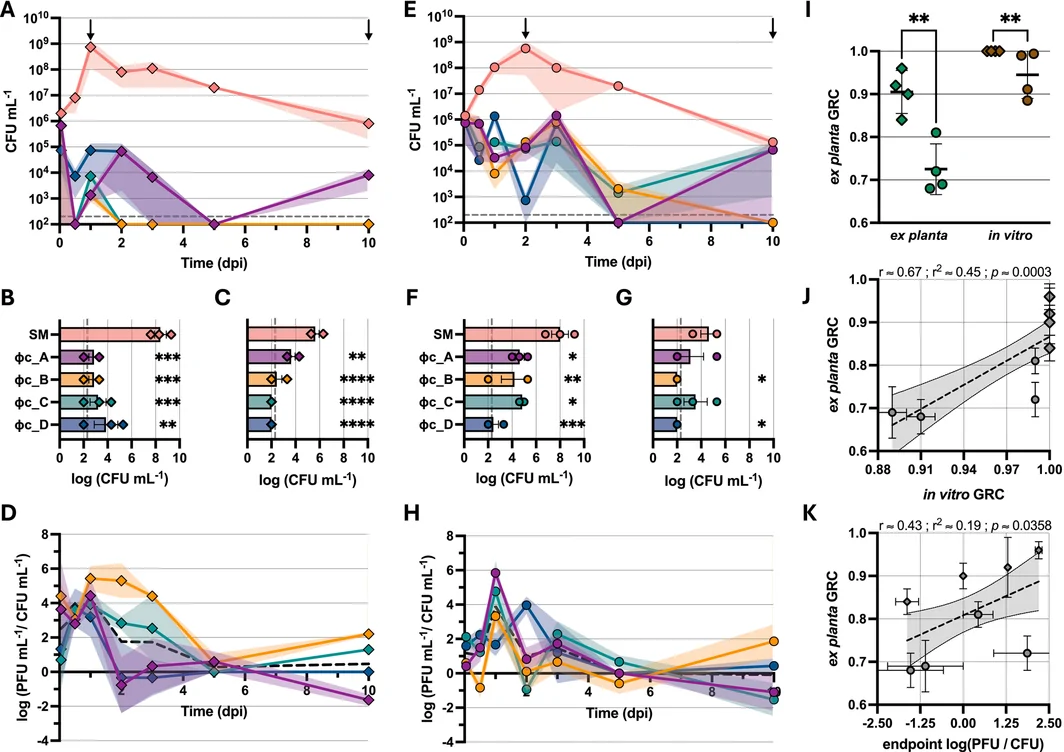
Phage cocktails suppress *Pseudomonas syringae* complex colonization of highbush blueberry leaves. Bacterial density over time (A, E) and at early (1 dpi; B, F) and late (10 dpi; C, G) timepoints, along with phage‐to‐bacteria ratios over time (D, H) for *P. syringae* complex strains B11 (diamonds; A–D) and B04 (circles; E–H) colonizing detached highbush blueberry leaves pretreated with SM (red) or phage cocktails φ c_A (violet), φc_B (orange), φc_C (teal) or φc_D (blue). Comparison of phage cocktail treatment efficacy estimated using Growth Reduction Coefficients (GRCs) ex planta (green) and in vitro (brown) for strains B11 and B04 (I). Correlations between ex planta GRCs and in vitro GRCs (J) and ex planta endpoint phage‐to‐bacteria ratios (K) for all tested phage cocktails. All points show the means of three independent biological replicates, while error bars and non‐bordered shaded regions show SEM. Black arrows in (A) and (E) highlight the early (1 dpi) and late (10 dpi) comparison timepoints respectively shown in (B), (F) and (C), (G). Light grey dashed lines in (A–C, E–G) demarcate the limit of detection (LOD), and a value equivalent to LOD/2 was assigned in cases where bacterial concentrations fell below this limit. Heavy black dashed lines in (D) and (H) show the mean PFU/CFU value trend across all phage cocktails, while light black dashes in Panels (J) and (K) trace the best‐fit lines, for which the correlation coefficients (*r*), coefficients of determination (*r* ^2^) and significances of slope deviation from zero (*p*) are provided above the panels, and the 90% confidence intervals of these lines are shown as bordered shaded regions. Differences between groups in (B), (C), (F), (G) and (I) were evaluated using Kruskal–Wallis tests followed by Dunn's post hoc multiple comparisons tests, with ****, ***, ** and * indicating *p* < 0.0001, *p* < 0.001, *p* < 0.01 and *p* < 0.05, respectively. For simplicity, only inter‐strain statistical comparisons are shown in Panel (I).

As a preliminary assessment of antibacterial activity, we next conducted planktonic killing (PK) assays with all eight individual phages (representative data in Figure 5A–D, all data in Figure S2) targeting a panel of eight Psc strains previously shown to be virulent in *V. corymbosum* (Latchman et al. 2026a, 2026b; also see Table S1). PK data were subsequently summarized for all phage‐host pairs (*n* = 64) using the Growth Reduction Coefficient (GRC; Figure 6E–H), and phages were ranked in overall antibacterial efficacy based on their median GRCs across all strains (left side of Figure 6G). Since previous work in other organisms has shown that the efficacies of certain ‘strong’ phages can be further improved through combinations with phages that are ineffective on their own (Niu et al. 2021; Lauman and Dennis 2023; Kaneko et al. 2026), our two most effective phages were variously combined with the four least effective phages to form eight unique phage cocktails (φC; see Table S3 for details), which were then assessed for antibacterial efficacy against all eight Psc strains (*n* = 64; all data in Figure S3) using the same approach. In general, planktonic Psc phage‐host or cocktail‐host interactions can be categorized as those where bacterial growth is abolished (A; GRC ≥ 0.97; Figure 5A), temporarily suppressed (B; 0.97 > GRC > 0.2; Figure 5B), unaffected (C; 0.2 ≥ GRC ≥ 0.0; Figure 5C) or improved (D; GRC < 0.0; Figure 5D) by phage(s). In terms of their potential suitability for applications in biocontrol, phages and phage cocktails can be deemed strong (GRC > 0.75), acceptable (0.75 ≥ GRC ≥ 0.5) or poor (GRC < 0.5) against a particular strain of interest, or broadly across all tested strains in a host library.

Among single‐phage treatments, 96.9% of interactions fell into categories A (*n* = 14; 21.9%) B (*n* = 33; 51.6%) or C (*n* = 15; 23.4%), while adverse outcomes (category D; *n* = 2; 3.1%), although possible, were rare (Figure 5E). Concordantly, the median GRC across all 64 pairs was only 0.47, with roughly 51.6% of phage‐host interactions rated as poor (GRC < 0.5), 7.8% as acceptable (0.75 ≥ GRC ≥ 0.5), and only 40.6% deemed strong (GRC > 0.75). Due to the universally broad and often multimodal distributions of per phage GRC scores (Figure 5G), few significant comparisons can be made between individual phages based on their median GRCs. Notably, however, the jumbo myovirus φCB10 had the highest median GRC (0.943) and the highest number of category A interactions (4 out of 8), and is the only phage in our panel that can be rated as strong across all targeted host strains. Podovirus φCB05 (median GRC = 0.620) was the only phage deemed acceptable, while all our other phages were rated as poor when judged across the entire host library (Figure 5E,G). Nevertheless, even phages with the lowest overall efficacies exhibited growth suppression capacities deemed acceptable or strong against certain host strains, suggesting that antibacterial efficacy is highly dependent on the specific bacterial host. Indeed, since GRC scores were distributed at least bimodally for almost every phage (Figure 5G) but were distributed more unimodally when categorized by strain (Figure 5H), we surmise that variation in antibacterial efficacy was driven more substantially by differences between strains than by differences between phages. Strains B11, B04 and B08 appeared hypersusceptible to phage killing in general, for instance, with nearly 43% of category A interactions accounted for by pairings involving B11. In contrast, all ineffective (C) or adverse (D) interactions occurred in phage‐host pairs containing strains B10, B09 or B12, suggesting that these strains are broadly unsusceptible to phage predation (Figure 5E,H). Interestingly, we observed modest but significant correlations between GRC and bacterial doubling time (*r* = 0.38; *p* = 0.0022; Figure S4A) and semiquantitative phage‐host interaction score (*r* = 0.43; *p* = 0.0004; Figure S4B), suggesting that both host growth rate and infection compatibility are predictors of planktonic growth suppression.

Among interventions with φCs, interaction distributions shifted slightly in favour of ‘extreme’ categories A (*n* = 18; 28.1%) and D (*n* = 5; 7.8%), but the majority of interactions nevertheless fell into categories B (*n* = 27; 42.2%) or C (*n* = 14; 21.9%). In line with this shift, the median GRC across all 64 pairs increased to 0.561, with 46.9% of interactions rated as poor and 45.3% as strong, while the proportion of interactions deemed acceptable remained 7.8% (Figure 5F). Critically, although φCs were significantly more effective than single phages against strain B12 (*p* < 0.0001), they were simultaneously less effective than single phages against B08 (*p* < 0.0001) and B05 (*p* = 0.0088; Figure 5H) and the difference between the overall GRC distributions for individual phages and φCs was not significant (Figure S5A). Predictably, differences between GRC distributions for individual phages and φCs were also insignificant among the most hypersensitive (B11, B04) and broadly unsusceptible (B10, B09) Psc strains, suggesting that combining strong and weak individual phages has no effect on suppressing the growth of these strains (Figure 5H). φC GRC distributions were unimodal and relatively narrow on all Psc strains except B06, thereby reinforcing the importance of strain effects. In contrast, GRC distributions per cocktail were invariably wide and multimodal, echoing our observations for individual phages (Figure 5G). The composition of our φCs ranged between three and five phage variants (Table S3), but the number of variants had no significant effect on the GRC distributions of φCs (Figure S5B). Although none of our φCs achieved median GRCs surpassing that exhibited by myovirus φCB10 alone, φC_A (median GRC = 0.895), φC_B (0.844), φC_C (0.782) and φC_D (0.778), named according to median GRC rank, were rated as strong across all targeted Psc strains (Figure 5G), and were selected for downstream applications ex planta.

### 3.5 Phage Cocktails Suppress *P. syringae* Complex Colonization of Highbush Blueberry Leaves

To gauge the potential utility of our φCs for agricultural biocontrol of the Psc, we tested the efficacy of prophylactic treatment with our four best φCs on reduction of Psc colonization of detached highbush blueberry leaves. Strains B11 (Figure 6A–D) and B04 (Figure 6E–H), selected based on their high virulence in *V. corymbosum* (Latchman et al. 2026b) and in vitro susceptibility to phages (Figure 5H), reached maximal densities in untreated leaves after one and 2 days post infection (dpi), respectively, and bacterial densities subsequently decreased over time (Figure 6A,E). In general, bacterial densities were decreased substantially in all leaves pretreated with φCs, and the short and long‐term biocontrol capacities of individual cocktails were assessed through comparisons at specific timepoints. At early timepoints, corresponding to maximal densities in untreated leaves, all four φCs induced significant bacterial density reductions ranging between 4.5 and 5.5 logs (all 0.05 > *p* ≥ 0.0004) when targeting B11 (Figure 6B), and between 3.2 and 5.6 logs (all 0.05 > *p* ≥ 0.0004) when targeting B04 (Figure 6F). Similar trends were observed at late timepoints corresponding to the experimental endpoint 10 dpi, suggesting that when effective our cocktails have potential for longitudinal suppression of Psc colonization of highbush blueberry leaves. Indeed, φC_C and φC_D reduced endpoint B11 densities to below the detection limit (both *p* < 0.0001), while φC_B reduced the titre of this strain by roughly 3.2 logs (*p* < 0.0001) and reduction by φC_A was more modest (2 logs; *p* = 0.002; Figure 6C), but only φC_B and φC_D significantly reduced the endpoint density of B04, to below the limit of detection (both *p* = 0.0408; Figure 6G).

To comprehensively quantify the ex planta antibacterial effects of our φCs across the entire experimental period in a manner that permits straightforward comparison with our findings in vitro, we analysed the bacterial density curves from our detached‐leaf infection assays using a variant of the GRC approach. All four φCs were deemed strong (GRC > 0.75) when targeting B11, and φC_D was also strong against B04 while the effects of the other three φCs were rated as acceptable (0.75 ≥ GRC ≥ 0.50) on this strain (Figure 6I). Importantly, the phage susceptibilities of these two strains ex planta appear to mirror those observed previously in vitro. Indeed, B11 is more susceptible than B04 to φC‐mediated growth inhibition both in vitro and ex planta (both *p* = 0.0013; Figure 6I) and, critically, ex planta GRCs correlate strongly (*r* = 0.67; *p* = 0.0003) with in vitro GRCs on the level of individual φCs (Figure 6J)—suggesting that φC performance in vitro is at least partially predictive of antibacterial efficacy ex planta. Nevertheless, ex planta GRCs were universally lower than their in vitro counter parts (*p* = 0.0001 in both strains, when comparing means; Figure 6I) indicating that complete bacterial eradication may not be achievable ex planta.

To confirm that reductions in bacterial density result from the lytic replication of our φCs, we collected PFU counts from infected leaves (Figure S6) and computed in situ multiplicities of infection (MOIs) at all experimental timepoints. Targeting the more susceptible strain B11, the mean in situ MOI of our φCs rose rapidly to a maximum of ≈1.8 × 10^4^ PFU mL^−1^/CFU mL^−1^ at 1 dpi, before gradually decaying to roughly 3 × 10^0^ PFU mL^−1^/CFU mL^−1^ by 10 dpi (Figure 6D). Mean in situ MOI for φCs targeting B04 similarly peaked at ≈7.9 × 10^3^ PFU mL^−1^/CFU mL^−1^ at 1 dpi and subsequently fell just below 1 × 10^0^ PFU mL^−1^/CFU mL^−1^ by the experimental endpoint (Figure 6H). Although assessment of in situ MOI trends for individual cocktails across the entire infection period remains challenging, a modest positive correlation (*r* = 0.43; *p* = 0.0358) was observed between endpoint in situ MOI and ex planta GRC despite our small sample size (Figure 6K). Collectively, these data are consistent with reduction of bacterial density ex planta being driven by the replication of lytic bacteriophages in our φCs.

## 4 Discussion

The *Pseudomonas syringae* complex (Psc) remains a significant threat to agricultural productivity worldwide, yet despite growing interest in phage‐based agricultural biocontrol (PhAB) strategies, no previous studies have examined the use of bacteriophages to specifically target blueberry‐associated strains of this phytopathogen. Here, we applied a multi‐stage screening pipeline to identify candidate phages suitable for agricultural biocontrol of Psc populations recovered from highbush blueberry plants throughout southwestern British Columbia (Figure 1). Initial host‐range screening revealed substantial variation in infectivity among phage isolates, and a semi‐quantitative approach was therefore utilized to identify candidates exhibiting both broad host ranges and high infectivity (Figure 2). Comparative genomic analyses of selected candidates subsequently uncovered considerable phylogenetic diversity and facilitated the exclusion of redundant or potentially unsuitable isolates (Figure 3 and Table 1). Environmental stability assays verified that candidate phages generally retained infectivity under conditions relevant to agricultural deployment (Figure 4 and Table 2), and in vitro antibacterial efficacy testing then identified the most broadly effective bacteriophages and enabled the selection of phages for cocktail formulation (Figure 5). Importantly, cocktails assembled using this approach were capable of longitudinally suppressing epiphytic Psc colonization of detached highbush blueberry leaves (Figure 6), demonstrating efficacy in a biologically relevant ex planta model. Collectively, these findings establish a framework for the systematic identification and evaluation of candidate phages for agricultural biocontrol, and demonstrate that phage cocktails can effectively suppress blueberry‐associated Psc populations under controlled experimental conditions.

Among the most informative outcomes of our screening pipeline were the relationships between static phage‐host interaction scores, in vitro planktonic killing efficacy and longitudinal suppression of bacterial colonization in the ex planta model. Semi‐quantitative interaction scores were positively correlated with planktonic growth suppression in vitro, suggesting that phages capable of producing stronger infections on solid medium may also exhibit greater antibacterial activity in liquid culture. However, this relationship was asymmetrical: low interaction scores were almost invariably associated with poor bacterial growth suppression, whereas high interaction scores corresponded to a much broader range of outcomes. Similar observations have been reported in previous systematic studies of phage host interactions (Haines et al. 2021; Lauman and Dennis 2023), implying that successful infection is necessary but not sufficient for effective bacterial control. Notably, planktonic growth reduction scores were distributed narrowly when organized by strain but had much broader, often multimodal distributions when organized by phage, suggesting that variation in the efficacy of planktonic growth suppression is influenced more critically by properties intrinsic to bacterial hosts than by those intrinsic to phages. Bacterial doubling time was modestly correlated with in vitro growth reduction, suggesting that bacterial growth dynamics may play an important role, but a complete understanding of how host characteristics impact growth suppression by phages nevertheless remains elusive and warrants further investigation. Importantly, antibacterial efficacy observed in planktonic assays was predictive of performance in the detached‐leaf model, and the relative susceptibility of individual strains was conserved between in vitro and ex planta systems. The predictive value of in vitro killing assays observed in the present study is particularly encouraging given that a similar relationship was recently observed between in vitro and in vivo outcomes in at least one other phage biocontrol system (Lauman, Hussain, et al. 2026). Together, these findings provide support for the use of tiered screening pipelines and suggest that planktonic killing assays may serve as a useful proxy for more complex validation models in high‐throughput screens.

Although ex planta efficacy varied among formulations and host strains, all tested cocktails produced appreciable reductions in bacterial burden when evaluated using the ex planta GRC framework, with most treatments meeting criteria for strong suppression. Curiously, the finding that substantial suppression of bacterial colonization was achieved through several distinct formulations suggests that effective cocktail design may be relatively tolerant of at least modest differences in cocktail composition. Crucially, treatment efficacy was positively associated with phage abundance relative to bacterial abundance at experimental endpoints, which is consistent with the hypothesis that bacterial suppression was mediated by ongoing phage replication within the leaf environment. While causality cannot be established from these data alone, the relationship between phage: bacterium dynamics and treatment outcome implies that successful biocontrol may depend not only on the killing efficiency of individual phages, but also on their capacity to persist within the plant environment and maintain productive infections over extended periods of time (Balogh et al. 2010; Iriarte et al. 2012; Buttimer et al. 2017). These findings demonstrate that phage‐mediated suppression of blueberry‐associated Psc can be sustained in a biologically relevant model system and thus support the continued development of phage‐mediated treatments for agricultural deployment.

The results of our genomic and environmental stability analyses emphasize the importance of evaluating biocontrol candidate phages on the basis of criteria beyond antibacterial efficacy. Comparative genomics revealed substantial phylogenetic diversity among isolates recovered from a relatively restricted geographical region, reiterating the notion that natural environments are rich reservoirs of potential biocontrol agents (Clokie et al. 2011; Howard‐Varona et al. 2017; Holtappels et al. 2021), while simultaneously identifying multiple clonal groups that were ultimately collapsed into representative lineages. These findings highlight the value of phage genome sequencing not only as a taxonomic tool, but more importantly as a means of avoiding redundancy during cocktail development and ensuring that candidate collections capture meaningful biological diversity. Critically, all sequenced lineages were predicted to be OL and lacked identifiable lysogen‐associated genes, antimicrobial resistance determinants or metal resistance cassettes, characteristics which are generally deemed indispensable for PhAB (Skurnik and Strauch 2006; Cook and Hynes 2025). Environmental stability assays likewise uncovered considerable heterogeneity among tested phages, with certain isolates exhibiting pronounced sensitivity to specific stresses while others remained stable across a broad range of conditions. Particularly striking was the apparent partitioning of freezing tolerance according to predicted virion morphology, with podovirus‐like phages generally exhibiting substantially greater stability than predicted myoviruses at freezing temperatures. This suggests that the structural and morphological properties of virions may broadly influence their environmental persistence, and future investigations into the mechanistic underpinnings of this phenomenon are therefore highly warranted. Notably, prolonged exposure to highly acidic conditions was among the most consistently destabilizing stresses, a potentially relevant finding given that blueberry plants exhibit optimum viability in acidic soil at pH 4.0–5.5 (Caspersen et al. 2016; Yang et al. 2022). Nevertheless, reductions in viability typically occurred only after extended incubations, suggesting that most phages may retain functionality in acidic environments over biologically relevant timescales, particularly in the presence of their bacterial hosts. Despite substantial variation in individual stability profiles, most phages remained stable under intermediate temperatures and irradiation levels likely to be encountered during agricultural deployment in British Columbia, and retained infectivity for prolonged periods even under ultimately destabilizing conditions.

Among the phages characterized in this study, φCB10 emerged as a particularly promising candidate for future development, consistently ranking among the strongest performers across multiple stages of the screening pipeline. Although the host range of this phage was not the broadest observed, φCB10 exhibited exceptionally strong antibacterial activity in planktonic killing assays and was the only phage candidate to achieve a median growth reduction score corresponding to strong suppression across all tested hosts. Moreover, cocktail formulations containing φCB10 demonstrated the strongest and most consistent efficacy across both ex planta host strains, further suggesting that this phage may contribute meaningfully to bacterial suppression in more complex biological systems. In addition to its efficacy, φCB10 also exhibited favourable stability under several agriculturally relevant conditions, including ultraviolet exposure and prolonged incubation in acidic conditions, characteristics that may prove advantageous for deployment in blueberry production systems (Iriarte et al. 2007; Caspersen et al. 2016; Yang et al. 2022). Importantly, the potential utility of φCB10 is further reinforced by its status as a jumbo phage. Jumbo phages remain comparatively underexplored relative to their conventional counterparts, yet are increasingly recognized as promising biocontrol agents due to their large coding capacities and frequent encoding of accessory functions capable of circumventing bacterial defence systems, including restriction‐modification, CRISPR‐Cas, and other anti‐phage mechanisms (Yuan and Gao 2017; Naknaen et al. 2024; Paranos et al. 2025; Yuping et al. 2025). Indeed, the substantial proportion of hypothetical proteins encoded by this phage suggests that it may possess a diverse repertoire of as yet uncharacterized biological functions (Hatfull 2015). Finally, the extreme divergence of φCB10 from currently described jumbo phages raises the tantalizing possibility that this phage may represent the founding member of a novel genus, further underscoring its biological and applied significance. Collectively, these findings identify φCB10 as an especially compelling subject for future investigations.

Although the results of this study support the feasibility of phage‐mediated biocontrol of blueberry‐associated Psc populations, several important questions remain to be addressed before such approaches may be translated into practical agricultural applications. Most notably, while detached‐leaf assays provide a biologically relevant intermediate between in vitro screening and field deployment, particularly in the context of controlling epiphytic Psc populations that contribute to blueberry leaf blight, they nevertheless cannot fully recapitulate the complexity of whole‐plant systems in which environmental variability, plant physiology and microbial community interactions collectively influence treatment outcomes (Balogh et al. 2010; Buttimer et al. 2017; Signorini et al. 2023). Given the exploratory nature of these experiments, a critical next step will therefore involve determining whether the efficacy reported here can be reproduced in larger‐scale studies using intact plants and, ultimately, under field conditions. Simultaneously, these future studies should investigate whether phage application may otherwise impact plant health or the integrity of the native phyllosphere microbiome. Further progress will also require a more complete understanding of the factors governing phage susceptibility in Psc populations. The correlations observed between host growth characteristics, static phage‐host interaction scores, and planktonic antibacterial efficacy suggest that bacterial traits play important roles in determining treatment outcomes, yet the specific genetic and physiological mechanisms underlying these relationships remain poorly understood. Integrating comparative host genomics with targeted studies of phage receptors and resistance mechanisms may therefore provide valuable insights into the determinants of successful phage‐mediated control. Finally, the exceptional performance of jumbo phage φCB10 identifies this lineage as a particularly attractive target for future investigations. Detailed characterization of the genome of this phage, its interactions with bacterial defence systems, receptor usage, and potential to steer bacterial populations towards less virulent phenotypes will facilitate its development as a biocontrol agent while simultaneously advancing our broader understanding of jumbo phage biology (Yuan and Gao 2017; Gurney et al. 2020; Supina et al. 2025). These avenues of research will bridge the gap between laboratory‐scale screening and practical deployment of phage‐based interventions in blueberry production systems and beyond.

This study provides the first systematic evaluation of bacteriophages targeting blueberry‐associated Psc populations and demonstrates that these viruses are promising candidates for the biocontrol of this problematic pathogen. By integrating host‐range analysis, comparative genomics, environmental stability testing, in vitro antibacterial efficacy assays and ex planta validation into a unified screening pipeline, we identified phage candidates exhibiting characteristics desirable for agricultural deployment and assembled cocktail formulations capable of longitudinal suppression of Psc colonization on detached highbush blueberry leaves. These findings demonstrate that the suitability of a phage for biocontrol is influenced by factors extending beyond antibacterial efficacy alone, and underscore the importance of evaluating candidate phages across multiple biological and environmental contexts. Finally, the identification of φCB10 as an unusually effective and biologically intriguing candidate highlights the value of continued exploration of undercharacterized phage lineages for applications in agricultural biocontrol. Taken together, these findings establish a foundation for the development of phage‐based interventions targeting highbush blueberry‐associated Psc populations, and contribute to the growing body of evidence supporting the use of bacteriophages as a sustainable and efficacious alternative to conventional antimicrobial strategies.

## Author Contributions

**Cassidy Ball:** writing – original draft, formal analysis, investigation, data curation. **Philip Lauman:** writing – review and editing, visualization, formal analysis, validation. **Tongzhou Xu:** software, writing – review and editing. **Thomas Guy:** software, writing – review and editing. **Kasia Dadej:** resources. **Robin Richter:** resources. **Mark Lubberts:** software. **Keiran Cross:** formal analysis. **Meilin Ren:** formal analysis. **Someshwar Ravnit Latchman:** methodology, data curation. **Rishi Burlakoti:** methodology, resources, writing – review and editing. **Xiangyu Deng:** resources, software. **Karen Fong:** writing – review and editing, funding acquisition, resources. **Siyun Wang:** writing – review and editing, conceptualization, funding acquisition, supervision, project administration.

## Funding

This work was supported by Genome British Columbia (GIR007).

## Conflicts of Interest

The authors declare no conflicts of interest.

## Supporting information


**Figure S1:** Correlations between number and strength of interactions. Scatterplots comparing the total number of interactions against the proportion of those interactions that are strong, per phage (A) and per bacterial strain (B). Heavy dashes trace best‐fit lines, for which the correlation coefficient (*r*), coefficient of determination (*r* ^2^) and significance of slope deviation from zero (*p*) are provided above each panel, while the 95% confidence intervals of these lines are shown as shaded regions. Data presented herein are computed from phage‐host interaction scores presented in Figure 2, which are the means of two independent biological replicates.
**Figure S2:** Planktonic growth inhibition of *Pseudomonas syringae* complex by individual phages. Growth curves of Psc strains B04 (A), B05 (B), B06 (C), B08 (D), B09 (E), B10 (F), B11 (G) and B12 (H) treated with SM (red) or phages φCB01 (light blue), φCB02 (blue), φCB03 (dark blue), φCB05 (purple), φCB08 (brown), φCB10 (teal), φCB11 (yellow) and φCB16 (orange). Lines and shaded regions respectively show the means and SD of at least three independent biological replicates.
**Figure S3:** Planktonic growth inhibition of *Pseudomonas syringae* complex by phage cocktails. Growth curves of Psc strains B04 (A), B05 (B), B06 (C), B08 (D), B09 (E), B10 (F), B11 (G) and B12 (H) treated with SM (red) or phage cocktails φC_A (light blue), φC_B (blue), φC_C (dark blue), φC_D (purple), φC_E (brown), φC_F (teal), φC_G (yellow) and φC_H (orange). Lines and shaded regions respectively show the means and SD of at least three independent biological replicates.
**Figure S4:** Correlations between host factors and growth reduction coefficient. Scatterplots comparing bacterial doubling time (A), and infectivity by specific phages, measured using the phage‐host interaction score (B), against the Growth Reduction Coefficients (GRCs) of those phages. Points in Panel (A) show the mean values obtained per host across all eight individual phage treatments (also see Figure 5), while error bars show SD of those replicates. Points in Panel (B) show phage‐host interaction scores presented in Figure 2 and individual phage GRCs shown in Figure 5, which are the means of two and three independent biological replicates, respectively. Heavy dashes trace the best‐fit lines, for which the correlation coefficients (*r*), coefficients of determination (*r* ^2^) and significances of slope deviation from zero (*p*) are provided above each panel, while the shaded regions show the 95% confidence intervals of these lines.
**Figure S5:** Efficacy comparisons for cocktails and individual phages. Comparisons of Growth Reduction Coefficients (GRCs) between individual phages (dark blue) and phage cocktails (teal; A), and between cocktails containing different numbers of phage variants (shades of grey, B). Individual points represent the means of at least three independent biological replicates, while the central bars and error bars respectively show the median and IQR for each group. Differences between groups in Panels (A) and (B) were respectively identified using Mann–Whitney and Kruskal–Wallis tests, with ns indicating *p* > 0.05.
**Figure S6:** Phage density in highbush blueberry leaves. In situ phage density over time in detached highbush blueberry leaves pretreated with phage cocktails φc_A (violet), φc_B (orange), φc_C (teal) or φc_D (blue), and colonized by *P. syringae* complex strains B11 (A) and B04 (B). All points show the means of three independent biological replicates while shaded regions show SEM. Light grey dashed lines demarcate the limit of detection (LOD), and a value equivalent to LOD/2 was assigned in cases where phage concentration fell below this limit.
**Table S1:** *Pseudomonas syringae* complex strains utilized in this study.
**Table S2:** *Pseudomonas* phages isolated in this study.
**Table S3:** Phage cocktails used in this study.
**Table S4:** Phage lifestyle predictions based on function‐associated CDSs.
**Table S5:** Phage morphology predictions based on function‐associated CDSs.

## Data Availability

The data that support the findings of this study are openly available in NCBI BioProject at https://www.ncbi.nlm.nih.gov/bioproject/PRJNA1477681/, reference number PRJNA1477681.
