# Supplementary material for: Bacteriophages Control Epiphytic Pseudomonas syringae Populations in Highbush Blueberry Leaves
Source: Microb Biotechnol. 2026 Sep 8;19(9):e70438. doi: 10.1111/1751-7915.70438 (PMC13554743; doi:10.1111/1751-7915.70438)
Supplement: Supplementary file 1 [file MBT2-19-e70438-s001.docx]

**Figure S1: Correlations between number and strength of interactions.** Scatterplots comparing the total number of interactions against the proportion of those interactions that are strong, per phage (**A**) and per bacterial strain (**B**). Heavy dashes trace best-fit lines, for which the correlation coefficient (r), coefficient of determination (r^2^) and significance of slope deviation from zero (p) are provided above each panel, while the 95% confidence intervals of these lines are shown as shaded regions. Data presented herein are computed from phage-host interaction scores presented in **Fig. 2**, which are the means of two independent biological replicates.

**Figure S2: Planktonic growth inhibition of Pseudomonas syringae complex by individual phages.** Growth curves of Psc strains B04 (**A**), B05 (**B**), B06 (**C**), B08 (**D**), B09 (**E**), B10 (**F**), B11 (**G**) and B12 (**H**) treated with SM (red) or phages $\varphi$CB01 (light blue), $\varphi$CB02 (blue), $\varphi$CB03 (dark blue), $\varphi$CB05 (purple), $\varphi$CB08 (brown), $\varphi$CB10 (teal), $\varphi$CB11 (yellow), and $\varphi$CB16 (orange). Lines and shaded regions respectively show the means and SD of at least three independent biological replicates.

**Figure S3: Planktonic growth inhibition of Pseudomonas syringae complex by phage cocktails.** Growth curves of Psc strains B04 (**A**), B05 (**B**), B06 (**C**), B08 (**D**), B09 (**E**), B10 (**F**), B11 (**G**) and B12 (**H**) treated with SM (red) or phage cocktails $\varphi$C_A (light blue), $\varphi$C_B (blue), $\varphi$C_C (dark blue), $\varphi$C_D (purple), $\varphi$C_E (brown), $\varphi$C_F (teal), $\varphi$C_G (yellow), and $\varphi$C_H (orange). Lines and shaded regions respectively show the means and SD of at least three independent biological replicates.

**Figure S4: Correlations between host factors and growth reduction coefficient.** Scatterplots comparing bacterial doubling time (**A**), and infectivity by specific phages, measured using the phage-host interaction score (**B**), against the Growth Reduction Coefficients (GRCs) of those phages. Points in panel **A** show the mean values obtained per host across all eight individual phage treatments (also see **Fig. 5**), while error bars show SD of those replicates. Points in panel **B** show phage-host interaction scores presented in **Fig. 2** and individual phage GRCs shown in **Fig. 5**, which are the means of two and three independent biological replicates, respectively. Heavy dashes trace the best-fit lines, for which the correlation coefficients (r), coefficients of determination (r^2^) and significances of slope deviation from zero (p) are provided above each panel, while the shaded regions show the 95% confidence intervals of these lines.

**Figure S5: Efficacy comparisons for cocktails and individual phages**. Comparisons of Growth Reduction Coefficients (GRCs) between individual phages (dark blue) and phage cocktails (teal; **A**), and between cocktails containing different numbers of phage variants (shades of gray, **B**). Individual points represent the means of at least three independent biological replicates, while the central bars and error bars respectively show the median and IQR for each group. Differences between groups in panels **A** and **B** were respectively identified using Mann-Whitney and Kruskal-Wallis tests, with ns indicating p > 0.05.

**Figure S6: Phage density in highbush blueberry leaves.** In situ phage density over time in detached highbush blueberry leaves pre-treated with phage cocktails $\varphi$c_A (violet), $\varphi$c_B (orange), $\varphi$c_C (teal), or $\varphi$c_D (blue), and colonized by P. syringae complex strains B11 (**A**) and B04 (**B**). All points show the means of three independent biological replicates while shaded regions show SEM. Light grey dashed lines demarcate the limit of detection (LOD), and a value equivalent to LOD/2 was assigned in cases where phage concentration fell below this limit.

**Table S1:** *Pseudomonas syringae* complex strains utilized in this study

| Shorthand ID | Full Strain Name | Species^a^ | Isolation Data | | | |
| --- | --- | --- | --- | --- | --- | --- |
|  |  |  | **Host** | **Variety** | **Location** | **Year** |
| B01^xx^ | BAB1.6C | *P. avellanae* | *Vaccinium sp*. | Unknown | Abbotsford | 2017 |
| B02^xx^ | BAB2.4A | *P. avellanae* | *Vaccinium sp*. | Unknown | Abbotsford | 2017 |
| B03^xx^ | BVI1.1B | *P. syringae* | *V. corymbosum* | Draper | Qualicum Beach | 2018 |
| B04^bc^ | BVI1.8A | *P. syringae* | *V. corymbosum* | Draper | Qualicum Beach | 2018 |
| B05^bx^ | BDR1.1L | *P. syringae* | *V. corymbosum* | Bluecrop | Mission | 2018 |
| B06^bx^ | X.EL.1.A | *P. viridiflava* | *V. corymbosum* | Elliot | Surrey | 2019 |
| B07^xx^ | D.DR.2.A | *P. syringae* | *V. corymbosum* | Draper | Chilliwack | 2019 |
| B08^bx^ | Q.DR.1.A | *P. viridiflava* | *V. corymbosum* | Draper | Abbotsford | 2019 |
| B09^bx^ | L.DU.2.A | *Pseudomonas* spp. | *V. corymbosum* | Duke | Yarrow | 2019 |
| B10^bx^ | 20.AB.NL.1.B | *P. syringae* | *Vaccinium spp*. cross | Northland | Delta | 2020 |
| B11^bc^ | 20.Z.AU.4.A | *P. viridiflava* | *V. corymbosum* | Aurora | Surrey | 2020 |
| B12^bx^ | 20.Z.AU.5.A | *P. avellanae* | *V. corymbosum* | Aurora | Surrey | 2020 |
| B13^xx^ | 23.Y.AU.1.BB | *Pseudomonas* spp. | *V. corymbosum* | Aurora | Surrey | 2023 |
| B14^xx^ | 23.Y.AU.2.BB | *Pseudomonas* spp. | *V. corymbosum* | Aurora | Surrey | 2023 |
| B15^xx^ | 23.Y.DR.2.AG | *Pseudomonas* spp. | *V. corymbosum* | Draper | Surrey | 2023 |
| B16^xx^ | 23.Y.DR.5.AG | *Pseudomonas* spp. | *V. corymbosum* | Draper | Surrey | 2023 |
| B17^xx^ | 23.AE.DR.1AG | *Pseudomonas* spp. | *V. corymbosum* | Draper | Delta | 2023 |
| B18^xx^ | 23.AE.DR.2LB | *Pseudomonas* spp. | *V. corymbosum* | Draper | Delta | 2023 |
| B19^xx^ | 23.Z.AU.1.SiB | *Pseudomonas* spp. | *V. corymbosum* | Aurora | Surrey | 2023 |
| B20^xx^ | 23.Z.AU.2.BB | *Pseudomonas* spp. | *V. corymbosum* | Aurora | Surrey | 2023 |

^a^ *Pseudomonas* strains isolated between 2017 and 2020 were mostly characterized to phylogenomic species (see Latchman et al., 2026a), while the phylogenomic species of strains isolated in 2023 currently remain unknown.

^b^ Strain selected for use in planktonic killing assays on the basis of virulence in *V. corymbosum*

^c^ Strain selected for use in detached leaf assays on the basis of virulence in *V. corymbosum* and *in vitro* phage susceptibility.

**Table S2:** *Pseudomonas* phages isolated in this study.

| Phage | Isolation Data | | | |
| --- | --- | --- | --- | --- |
|  | **Isolation Host** | **Source** | **Location** | **Date** |
| $\varphi$CB01^x^ | B07 | Wastewater | Langley | 09/2023 |
| $\varphi$CB02^x^ | B05 | Wastewater | Richmond | 09/2023 |
| $\varphi$CB03^x^ | B05 | Wastewater | Richmond | 09/2023 |
| $\varphi$CB04^b^ | B07 | Wastewater | Richmond | 09/2023 |
| $\varphi$CB05^x^ | B07 | Wastewater | Richmond | 09/2023 |
| $\varphi$CB06^b^ | B05 | Wastewater | Delta | 07/2023 |
| $\varphi$CB07^a^ | B10 | Wastewater | Richmond | 09/2023 |
| $\varphi$CB08^x^ | B10 | Wastewater | Richmond | 09/2023 |
| $\varphi$CB09^a^ | B09 | Wastewater | Kelowna | 06/2023 |
| $\varphi$CB10^x^ | B09 | Wastewater | Kelowna | 06/2023 |
| $\varphi$CB11^x^ | B03 | Wastewater | Kelowna | 06/2023 |
| $\varphi$CB12^a^ | B09 | Wastewater | Vernon | 06/2023 |
| $\varphi$CB13^a^ | B10 | Wastewater | Vernon | 06/2023 |
| $\varphi$CB14^a^ | B01 | Blight sample (*V. corymbosum*; Aurora) | Surrey | 06/2023 |
| $\varphi$CB15^a^ | B02 | Blight sample (*V. corymbosum*; Aurora) | Surrey | 06/2023 |
| $\varphi$CB16^x^ | B08 | Blight sample (*V. corymbosum*; Draper) | Delta | 06/2023 |
| $\varphi$CB17^b^ | B03 | Gall & blight sample (*V. corymbosum*; Draper) | Delta | 06/2023 |
| $\varphi$CB18^b^ | B05 | Gall & blight sample (*V. corymbosum*; Draper) | Delta | 06/2023 |
| $\varphi$CB19^a^ | B05 | Gall & blight sample (*V. corymbosum*; Draper) | Delta | 06/2023 |

^a^ Excluded from downstream characterization and antibacterial efficacy analyses due to designation as a weak specialist.

^b^ Excluded from downstream characterization and antibacterial efficacy analyses due to clonal or near-clonal similarity to another phage.

**Table S3:** Phage cocktails used in this study.

| Cocktail ID | Weighted Composition | # Phage Variants | # Phage Species | Median GRC  *in vitro* |
| --- | --- | --- | --- | --- |
| 𝜑C_A | 𝜑CB02 (0.2), 𝜑CB05 (0.2), 𝜑CB11 (0.2), 𝜑CB16 (0.4) | 4 | 3 | 0.895 |
| $\varphi$C_B | $\varphi$CB05 (0.2), $\varphi$CB10 (0.2), $\varphi$CB11 (0.2), $\varphi$CB16 (0.4) | 4 | 3 | 0.844 |
| 𝜑C_C | 𝜑CB05 (0.4), 𝜑CB11 (0.2), 𝜑CB16 (0.4) | 3 | 2 | 0.782 |
| $\varphi$C_D | $\varphi$CB10 (0.2), $\varphi$CB11 (0.2), $\varphi$CB16 (0.6) | 3 | 2 | 0.778 |
| 𝜑C_E | 𝜑CB02 (0.2), 𝜑CB05 (0.2), 𝜑CB10 (0.2), 𝜑CB11 (0.2), 𝜑CB16 (0.2) | 5 | 4 | 0.578 |
| $\varphi$C_F | $\varphi$CB02 (0.2), $\varphi$CB05 (0.4), $\varphi$CB11 (0.2), $\varphi$CB16 (0.2) | 4 | 3 | 0.383 |
| $\varphi$C_G | $\varphi$CB05 (0.4), $\varphi$CB08 (0.2), $\varphi$CB11 (0.2), $\varphi$CB16 (0.2) | 4 | 3 | 0.290 |
| $\varphi$C_H | $\varphi$CB02 (0.2), $\varphi$CB05 (0.6), $\varphi$CB08 (0.2) | 3 | 3 | 0.266 |

**Table S4:** Phage lifestyle predictions based on function-associated CDSs.

| Phage Isolate | Number of Function-Associated CDSs | | | | Predicted Lifestyle |
| --- | --- | --- | --- | --- | --- |
|  | Integrase | Excisionase | Repressor | Recombinase |  |
| $\varphi$CB01 | 0 | 0 | 0 | 0 | OL |
| $\varphi$CB02 | 0 | 0 | 0 | 0 | OL |
| $\varphi$CB03 | 0 | 0 | 0 | 0 | OL |
| $\varphi$CB04 | 0 | 0 | 0 | 0 | OL |
| $\varphi$CB05 | 0 | 0 | 0 | 0 | OL |
| $\varphi$CB06 | 0 | 0 | 0 | 0 | OL |
| $\varphi$CB08 | 0 | 0 | 0 | 0 | OL |
| $\varphi$CB10 | 0 | 0 | 0 | 0 | OL |
| $\varphi$CB11 | 0 | 0 | 0 | 0 | OL |
| $\varphi$CB16 | 0 | 0 | 0 | 0 | OL |
| $\varphi$CB17 | 0 | 0 | 0 | 0 | OL |
| $\varphi$CB18 | 0 | 0 | 0 | 0 | OL |

**Table S5:** Phage morphology predictions based on function-associated CDSs.

| Phage Isolate | Number of Function-Associated CDSs | | | | | | | | | Predicted Morphology |
| --- | --- | --- | --- | --- | --- | --- | --- | --- | --- | --- |
|  | Tail Sheath | Baseplate | Tail Tube | Tail Assembly | Tail Fiber  or Host Recognition | Internal Virion or Head | Head or Capsid | Packaging | Lysis |  |
| $\varphi$CB01 | 0 | 0 | 0 | 0 | 2 | 4 | 2 | 2 | 3 | Podovirus |
| $\varphi$CB02 | 0 | 0 | 0 | 0 | 1 | 4 | 2 | 2 | 3 | Podovirus |
| $\varphi$CB03 | 0 | 0 | 0 | 0 | 2 | 4 | 2 | 2 | 3 | Podovirus |
| $\varphi$CB04 | 0 | 0 | 0 | 0 | 2 | 4 | 2 | 2 | 4 | Podovirus |
| $\varphi$CB05 | 0 | 0 | 0 | 0 | 2 | 5 | 2 | 2 | 5 | Podovirus |
| $\varphi$CB06 | 0 | 0 | 0 | 0 | 2 | 4 | 2 | 2 | 5 | Podovirus |
| $\varphi$CB08 | 0 | 0 | 0 | 0 | 2 | 5 | 2 | 2 | 5 | Podovirus |
| $\varphi$CB10 | 2 | 1 | 0 | 1 | 3 | 11 | 3 | 1 | 2 | Myovirus |
| $\varphi$CB11 | 2 | 3 | 1 | 1 | 1 | 0 | 4 | 0 | 1 | Myovirus |
| $\varphi$CB16 | 2 | 3 | 1 | 1 | 1 | 0 | 4 | 0 | 1 | Myovirus |
| $\varphi$CB17 | 2 | 3 | 1 | 1 | 1 | 0 | 4 | 0 | 1 | Myovirus |
| $\varphi$CB18 | 2 | 3 | 1 | 1 | 1 | 0 | 4 | 0 | 1 | Myovirus |
